# CF10 Displayed Improved Activity Relative to 5-FU in a Mouse CRLM Model Under Conditions of Physiological Folate

**DOI:** 10.3390/cancers17172739

**Published:** 2025-08-23

**Authors:** Charles Chidi Okechukwu, Xue Ma, Wencheng Li, Ralph D’Agostino, Matthew G. Rees, Melissa M. Ronan, Jennifer A. Roth, William H. Gmeiner

**Affiliations:** 1 Department of Cancer Biology, Wake Forest University School of Medicine, Winston-Salem, NC 27157, USA; cokechuk@wakehealth.edu; 2 Department of Orthopedic Surgery, Wake Forest University School of Medicine, Winston-Salem, NC 27157, USA; xma@wakehealth.edu; 3 Department of Pathology, Wake Forest University School of Medicine, Winston-Salem, NC 27157, USA; wenli@wakehealth.edu; 4 Department of Public Health Sciences and Comprehensive Cancer Center, Wake Forest University School of Medicine, Winston-Salem, NC 27157, USA; rdagosti@wakehealth.edu; 5 Broad Institute of MIT and Harvard, Cambridge, MA 02142, USA; reesm@broadinstitute.org (M.G.R.); mronan@broadinstitute.org (M.M.R.); jroth@broadinstitute.org (J.A.R.)

**Keywords:** colorectal cancer, fluoropyrimidine, leucovorin, replication stress, thymidylate synthase, topoisomerase 1

## Abstract

Background/Objective: At least 25% of colorectal cancer (CRC) patients develop liver metastases (CRLM), and chemotherapeutic regimens based on the fluoropyrimidine (FP) drug 5-fluorouracil (5-FU) provide a survival advantage, but long-term survival is uncommon. The primary molecular target of FP drugs is thymidylate synthase (TS). Methods: A TS/Top1 dual-targeting cytotoxic mechanism for CF10/LV was confirmed by TS ternary complex detection by Western blot and by immunofluorescence detection of Top1 cleavage complexes. CF10/LV activated the ATR/Chk1 pathway consistent with enhanced replication stress and induced apoptosis. In vivo studies showed CF10 and CF10/LV eradicated liver metastasis in a CRLM model without scarring or weight loss, displaying therapeutic advantages relative to legacy FPs. Results: We demonstrated that a nanoscale FP polymer, CF10, displayed greater potency than expected based on FP content in part through more direct conversion to the TS-inhibitory metabolite, FdUMP. In this study, we tested CF10 for potency advantages relative to 5-FU and trifluorothymidine (TFT, the FP component of TAS-102) and confirmed a general potency advantage for CF10 in CRC cell lines in the Broad Institute PRISM screen. We demonstrated that this potency advantage is retained in CRC cells cultured with human-like folate levels and is enhanced by LV co-treatment to a similar extent as that by 5-FU. Our results confirm CF10 development proceeding as a CF10/LV combination. Mechanistically, CF10 cytotoxicity closely correlates with poisons of DNA topoisomerase 1 (Top1) in the PRISM screen relative to 5-FU and TFT. Conclusions: Our pre-clinical data support an early-phase clinical trial for CF10 for treating liver-metastatic CRC.

## Simple Summary

The paper introduces CF10, a nanoscale fluoropyrimidine polymer with enhanced potency against colorectal cancer liver metastases (CRLM) compared to traditional agents such as 5-FU and TAS-102. CF10 demonstrates a dual-action mechanism—thymidylate synthase inhibition and topoisomerase I poisoning—resulting in replication stress and apoptosis, even in aggressive tumor cells. CF10 potency was maintained under physiological folate levels and further boosted by leucovorin (LV) co-treatment. In vivo, CF10 and CF10/LV effectively eradicated liver metastases without toxicity. These findings support CF10/LV as a promising therapeutic strategy and set the stage for early-phase clinical trials, potentially reshaping treatment approaches in refractory CRC.

## 1. Introduction

The introduction of the fluoropyrimidine (FP) drug 5-fluorouracil (5-FU) into clinical use for the treatment of colorectal cancer (CRC) > 60 years ago was an inflection point, with 5-FU-based adjuvant chemotherapy improving outcomes in patients with localized disease and 5-FU-based regimens improving survival for patients with metastatic CRC (mCRC) [1]. The optimization of 5-FU-based chemotherapy through improved scheduling and the development of more effective combination therapy regimens have, in recent decades, substantially further improved outcomes, with mCRC patients now surviving an average of 32 months, during which many receive three or more lines of chemotherapy, each of which usually includes an FP drug (5-FU or trifluorothymidine (TFT), the FP component of TAS-102) [2,3,4]. Unfortunately, long-term survival remains uncommon for mCRC patients (<14% 5-year survival), and new therapeutic options are needed.

The proven activity of 5-FU-based chemotherapy regimens in mCRC has stimulated continued exploration of the mechanism responsible for antitumor activity and the development of next-generation FPs that further extend survival [5]. We previously reported that a second-generation FP polymer, CF10, displayed significantly greater cytotoxicity to CRC cells relative to 5-FU. Despite having only a 10-fold greater FP content, CF10 was >300-fold more potent than was 5-FU on average, based on GI50 values across all cell lines included in the NCI60 cell line screen [6]. Consistent with its strong clinical potential, we demonstrated that CF10 displayed improved antitumor activity relative to 5-FU in a mouse orthotopic model of primary colon cancer [6] and in a rat syngeneic orthotopic model of CRC liver metastasis (CRLM) [7]. CF10 also displayed reduced toxicity relative to 5-FU in both mouse and rat models, which is consistent with reduced conversion to ribonucleotide metabolites that contribute to GI-tract and hematological toxicities [8,9].

**Figure 1:**
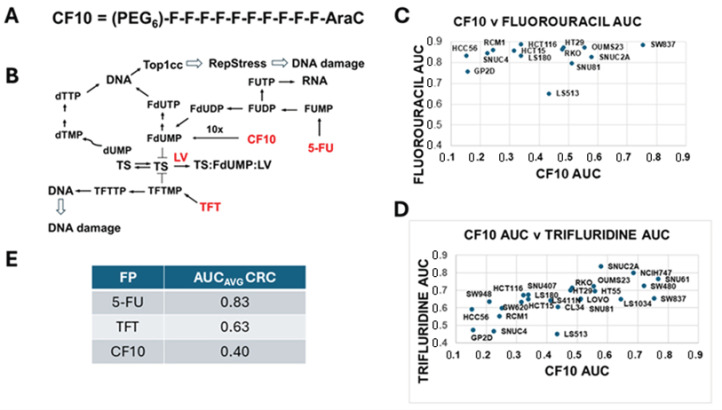
The activity of FPs converges on TS inhibition and DNA damage. (**A**) Structure of CF10 (PEG = polyethylene glycol, F = fluorodeoxyuridine, AraC = arabinosyl cytidine). (**B**) Scheme summarizing pathways for the three FPs included in the present study (CF10, 5-FU, TFT) that result in TS inhibition and cause DNA damage. (**C**,**D**) Plots of AUC for 5-FU vs. CF10 and for TFT vs. 5-FU, respectively. AUC values were from testing in the Broad Institute PRISM screen (data included in Supplementary Figure S2A–C). (**E**) Summary of AUC values from the Broad Institute PRISM screen for 5-FU, CF10, and TFT in *n* = 16 CRC cell lines in which all drugs were tested (data shown in Supplementary Tables S1–S3 and Figure S2).

The advancement of CF10 into clinical development requires establishing therapeutic advantages relative to legacy FPs (5-FU, TFT) in experimental models of CRC relevant to translation. In clinical regimens for mCRC, 5-FU is co-administered with leucovorin (LV). LV is converted to N5,N10-tetrahydrofolate, a reduced folate co-factor for TS which promotes the formation of a stable ternary complex consisting of FdUMP, the reduced folate co-factor, and the enzyme that irreversibly inhibits TS (Figure 1). In contrast, TFT which is used primarily in third-line treatment of mCRC inhibits TS without a requirement for a reduced folate co-factor [10], and TFT is used without LV in clinical regimens for mCRC. Whether CF10 development should proceed with LV co-treatment requires clarification before clinical studies are initiated. Since CF10 inhibits TS through the release of FdUMP, the same FP metabolite responsible for TS inhibition by 5-FU, CF10 would be anticipated to benefit from LV co-treatment. However, CF10 is cytotoxic at much lower concentrations than is 5-FU, and endogenous levels of reduced folates in cancer cells could be sufficient to promote ternary complex formation without LV co-treatment. Resolution of whether CF10 benefits from LV co-treatment is complicated by the fact that most media used to culture cancer cells contain supra-physiological folate levels [11]. One approach, which was adopted in these studies, is to perform assays in folate-controlled media to simulate human physiological levels [12].

While TS is recognized as a primary target for FPs (4) (Figure 1) the potency advantage of CF10 (Figure 1; Supplementary Figure S1) relative to legacy FPs results from factors other than TS inhibition. Insight into the mechanistic basis for CF10′s increased potency advantage relative to 5-FU came from a COMPARE analysis of data from the NCI60 cell line screen [13] that revealed the most closely related compounds were not TS inhibitors but DNA topoisomerase 1 (Top1) poisons (e.g., camptothecin) [14]. The mechanism by which CF10 poisons Top1 is distinct from CPTs and involves the misincorporation of FdUTP into genomic DNA under conditions of Thy depletion resulting from strong TS inhibition. In collaboration with Pommier, we showed that FdU inhibits the re-ligation step of Top1 catalysis, leading to accumulation of trapped Top1 cleavage complexes (Top1cc) proximal to sites of FdU substitution in DNA. Thus, CF10 is cytotoxic to CRC cells through dual targeting of TS and Top1 [15,16,17], resulting in Top1-mediated DNA double-strand breaks (DSBs) and enhanced replication stress [18,19]. In contrast, the COMPARE analysis did not demonstrate a strong correlation of 5-FU with Top1 poisons [15], significantly lower levels of Top1cc were detected in 5-FU-treated cancer cells, and these were reversed with Urd [18], consistent with an RNA-mediated process in their formation. While TFT is known to cause DNA damage [20], it has not been shown to cause Top1cc formation, and the increased potency of CF10 relative to TFT could result from CF10 causing increased DNA damage through a Top1-mediated process that is not relevant to the cytotoxic activity of TFT or 5-FU. Thus, the TS/Top1 dual-targeting mechanism of CF10 could differentiate CF10 from legacy FPs and result in the more effective treatment of mCRC and other malignancies, including effective treatment of disease that has progressed with prior FP treatment.

In this study, we tested CF10 and legacy FPs in additional CRC cell lines and under folate-restricted conditions to determine if CF10 is likely to provide an improved therapeutic response in clinical studies. Results from the Broad Institute PRISM screen confirmed a potency advantage for CF10 relative to 5-FU and TFT that was general across multiple CRC cell lines and consistent with broad-spectrum clinical activity. We also demonstrated that CF10 is more potent than 5-FU and TFT in CRC cells cultured with human-like folate levels and established that the improved potency of CF10 is enhanced with LV co-treatment to a similar extent as that with 5-FU, a result consistent with CF10 clinical development proceeding with LV co-treatment. Mechanistically, endpoints of TS inhibition, Top1cc formation, DNA damage, increased replication stress, and induction of apoptosis were established for CF10 and CF10+LV under conditions of folate restriction, providing a rationale for pharmacodynamic endpoint selection for clinical development. To test if the potency advantage for CF10 demonstrated in CRC cells results in improved antitumor activity, we simulated challenges associated with clinical CRLM treatment with FPs by (i) adapting mice to an FR diet to simulate human plasma folate levels [11]; (ii) testing antitumor activity in a syngeneic, orthotopic liver metastasis mouse model [7,21]; (iii) evaluating three FPs (CF10, 5-FU, TFT) under conditions that delivered equivalent FP amounts on a molar basis and via the same route to assess intrinsic anti-tumor activity; and (iv) including LV co-treatment [22]. We found that CF10 and CF10 + LV are highly potent in eradicating liver-metastatic CRC and that efficacy was achieved without weight loss or other signs of toxicity under conditions where equivalent levels of 5-FU and TFT offered less therapeutic benefit. Our studies indicate that CF10 and CF10/LV show promising activity for CRLM treatment under conditions that simulate human folate physiology and support CF10 clinical development.

## 2. Materials and Methods

### 2.1. Cell Lines, Reagents, and Clonogenic Assay

HCT116 (RRID:CVCL_0291), HCT15 (RRID:CVCL_0292), LS174T (RRID:CVCL_1384), and MC38 (RRID:CVCL_B288) colorectal cancer (CRC) cells were from ATCC and were cultured using recommended media or folate-restricted media (FR) [12], validated by short tandem repeat analysis, and regularly confirmed negative for Mycoplasma. CF10 was obtained from ST Pharma (Siheung-si, Korea), validated by high-resolution mass spectrometry, and dissolved in 0.9% sterile saline. Clinical-grade 5-FU (50 mg/mL) was purchased from the Baptist Hospital clinical pharmacy, while trifluridine (TFT) was purchased from MedChemExpress. 5-FU concentrations were calculated based on the dilution of the established stock concentration. CF10 concentrations were matched to deliver equivalent nucleoside content based on UV absorbance at 260 nm. A modified clonogenic assay assessed the potency of CF10, TFT, and 5-FU in CRC cells. CRC cells were plated in 24-well plates in both standard and folate-restricted media, and after 24 h, they were treated with the indicated concentration of CF10, TFT, or 5-FU for 72 h. The media were replaced after drug treatment, while the cells were allowed to grow for 168 h. Cell proliferation was evaluated using the Aqueous One (Promega, Madison, WI, USA) reagent, following the manufacturer’s instructions, after the cell solution was transferred to 96-well plates. Apoptosis was evaluated using Caspase 3/7-Glo reagent (Promega) following the manufacturer’s instructions.

### 2.2. Western Blotting

Proteins were isolated, and their differential expressions were analyzed by Western blot, as described previously [6]. Briefly, the cells were lysed using RIPA buffer (50 mM Tris HCl at pH 7.4, 150 mM NaCl, 1% Triton X-100 or NP-40, 0.5% sodium deoxycholate, 0.1% SDS, 1 mM EDTA, and 10 mM NaF freshly supplemented with protease and phosphatase inhibitors). The protein concentrations were quantified using a Bradford assay (Bio-Rad, Hercules, CA, USA), and the samples were normalized for equal loading. All samples were then heated to 100 °C for 10 min in the presence of 6× Laemmli buffer (Boston Bio-Products; Milford, MA, USA). SDS-PAGE resolved the proteins, and their expression was analyzed following immunoblotting using specific antibodies. The following antibodies were used in this study: Rad51 (Cell Signaling Technology, Danvers, MA, USA, Cat# 8875, RRID:AB_2721109), Chk2 (Thermo Fisher Scientific, Waltham, MA, USA, Cat# MA5-35294, RRID:AB_2849196), pCHK2-T68 (Thermo Fisher Scientific Cat# PA5-104715, RRID:AB_2816188); RPA32/RPA2 (Cell Signaling Technology Cat# 52448, RRID:AB_2750889), thymidylate synthase (Cell Signaling Technology Cat# 3766, RRID: AB_2210584), pRPA32-S33 (Cell Signaling Technology Cat# 10148, RRID:AB_3099645), Chk1 (Thermo Fisher Scientific Cat# MA1-23336, RRID:AB_558392); pCHK1-S317 (Cell Signaling Technology Cat# 2344, RRID:AB_331488), cleaved caspase 3-D175 (Cell Signaling Technology Cat# 9664, RRID:AB_2070042), and β-actin (Santa Cruz Biotechnology, Dallas, TX, USA, Cat# sc-47778 HRP, RRID:AB_2714189).

### 2.3. Cell Viability and Rescue Assay

HCT116, HCT15, LS174T, and MC38 cells were plated on 96-well, white, flat-bottom plates, and for 48 h, cells were allowed to adhere to the plate and begin to grow exponentially until they reached about 25% confluency. Exogenous uridine (Sigma, St. Louis, MO, USA) and thymidine (Sigma) were added to their designated wells. The plates were then shaken for 2 min by hand to mix appropriately without disrupting cell growth before being placed back in the incubator at 37 °C and with 5% CO_2_. After 48 h in the incubator, the medium was evacuated, and cell viability was determined using Promega kits as described elsewhere [6]. The rescue experiments were performed in triplicate with four data points in each experiment per tested condition.

### 2.4. TS Activity Assay

A TS in situ activity assay was performed as previously described [23] to provide a quantitative indication of intracellular inhibition of TS activity following drug treatment. Approximately ~0.5 × 10^6^ HCT-116 cells were plated in each well of 6-well plates, and cells were allowed to attach for 24 h and then treated with either 5-FU, 5-FU/LV, CF10, CF10/LV, TFT, or TFT/LV for 48 h. For the last 2 h, 0.16 Ci/mmol of [5-^3^H]-2′-deoxyuridine (Moravek, Brea, CA, USA) was present at a final concentration of 2.5 µM. Then, 150 µL of media from each well was added to an ice-cold suspension containing 750 µL of charcoal with 0.5% T-70 dextran and 2.5% BSA and 150 µL of 35% trichloroacetic acid. After centrifugation, a portion of the supernatant was counted by liquid scintillation counting, and enzyme activities were expressed as a percent relative to drug-free control.

### 2.5. Liver Tumor Cell Inoculation to Simulate CRLM Formation

All animal studies were undertaken in accordance with the protocols approved by the Institutional Animal Care and Use Committee at Wake Forest University School of Medicine (Winston-Salem, NC, USA). Immunocompetent C57BL6 (RRID:IMSR_JAX:000664) mice weighing 21–24 g were used because this strain is syngeneic with the MC38 colon carcinoma cell line. C57BL6 mice were commercially available in the U.S. and were obtained from Charles River Laboratories for these studies. The mice had a controlled climate and light cycles, and all had free access to a standard laboratory diet and water. For the inoculation procedure, anesthesia was obtained using 2–3% isoflurane and then maintained at 1.5% for surgery. A 2 cm midline incision was made, and both the left and right hepatic lobes were mobilized. The MC38 cells were suspended at a density of 2 × 10^6^ cells in 200 µL and 25 µL of cell suspension, mixed 1:1 with Matrigel, and injected via a 29-gauge needle into the subcapsular portion of one of the previously specified hepatic lobes. When the needle was withdrawn, a cotton swab was used to press on the puncture site to achieve hemostasis. Once hemostasis was achieved, the wound was washed with saline, the muscle layer was closed using a 6–0 Vicryl suture, and the skin was closed using staples. An abdominal bandage was applied, and the mice were allowed to recover from anesthesia before returning to the animal facility. The tumors were allowed to grow for a week and fluorescence imaging was performed using an RGD peptide Cy5.5 conjugate to establish tumor formation prior to the initiation of treatments [7].

### 2.6. Treatment and Fluorescence Imaging of Tumor Progression

The C57BL6/MC38 syngeneic orthotopic mouse model fluorescence tumor images were undertaken using an IVIS Lumina LT Series III system under isoflurane anesthesia. All images were analyzed using Living Image software (v4.7.4.) with an identical size region of interest for each mouse. To assess therapeutic efficacy, groups of *n* = 4 mice with similar initial tumor levels based on fluorescence imaging were treated with (1) no treatment, (2) 5-FU, (3) CF10, (4) TFT, (5) LV, (6) 5-FU+LV, (7) CF10+LV, or (8) TFT+LV. 5-FU was administered at 100 mg/kg [24], and TFT and CF10 were dosed to deliver equivalent nucleoside content based on UV absorbance at 260 nm. Leucovorin was infused together with the FP [25]. Mice in each group were treated using a 7-day mini-osmotic pump. All mice underwent a final imaging procedure at the end of the seventh day and were then euthanized, and their vital organs (livers, etc.) were extracted and prepped for other procedures. For each of the eight treatments, the difference in flux or weight values were calculated (Day 7 minus Day 0). Next, a general linear model was fit to compare the 8 groups (Control, 5FU, LV, CF10, TFT, 5FU + LV, CF10 + LV, TFT + LV). We first examined the overall test for the model, and if that was significant, a pairwise comparison between groups were made. Given the large number of potential pairwise comparisons that could be made, we conservatively used Bonferroni correction when comparing groups. The adjusted *p*-value for significance using a Bonferroni correction was 0.017 (0.05/3). For maintaining mice on a folate-restricted (FR) diet, the FR diet purchased from Envigo Teklad Diets (Madison, WI, USA) was composed of 35.0 g/kg of AIN-93G-MX (94046), 10.0 g/kg of succinylsulfathiazole, 195.0 g/kg of casein, 3.0 g/kg of L-cystine, 304.488 g/kg of corn starch, 0.016 g/kg of calcium pantothenate, 0.006 g/kg of thiamin (81%), 209.749 g/kg of sucrose, 130.0g/kg of maltodextrin, 60.0 g/kg of soybean oil, 50.0 g/kg of cellulose, 0.007 g/kg of pyridoxine HCl, 0.012 g/kg of TBHQ (antioxidant), 2.5 g/kg of choline bitartrate, 0.03 g/kg of niacin, 0.006 g/kg of riboflavin, 0.0002 g/kg of biotin, 0.025 g/kg of vitamin B12 (0.1% in mannitol), 0.0008 g/kg of vitamin K1 phylloquinone, 0.15 g/kg of vitamin E, DL-alpha tocopheryl acetate (500 IU/g), 0.008 g/kg of vitamin A palmitate (500,000 IU/g), and 0.002 g/kg of vitamin D3 cholecalciferol (500,000 IU/g).

### 2.7. PRISM Assay

CF10 was screened in 873 PRISM DNA-barcoded cell lines established by the Broad Institute. In brief, 20–25 cell lines per pool were plated in 384-well plates and treated with CF10 at 8 doses in three-fold dilutions starting at 9.6 μM for 5 days. Two PRISM cell line collections were used in the assay: PR500 (including only adherent cell lines) and PR300+ (including adherent and suspension cell lines). Cells were then lysed in TCL mRNA lysis buffer, and then PCR with reverse transcription was performed. Detection of the barcodes and univariate and multivariate analysis were then performed as previously described [26]. Benchmark test agents were also tested at dose to ensure high data quality. All test agents were run in triplicate, and each plate contained positive (bortezomib at 20 µM) and negative (DMSO) controls. Comparisons of drug potency between CF10 and other drugs tested in the PRISM screen were made based on the area under the curve (AUC), a metric in which more potent drugs display lower values reflecting decreased cell viability across the range of drug concentrations tested [27].

### 2.8. Statistical Analysis

GraphPad Prism 10.2.0 was used for statistical analyses of cell-based assays. Experiments were conducted in triplicate ± SEM. For all in vitro experiments, four biological replicates were used for analysis, and appropriate one-way ANOVA tests with recommended Tukey corrections were conducted, with *p* < 0.05 indicating significance. For in vivo studies, two one-way analysis of variance (ANOVA) models were fit to compare the 8 groups on the outcomes of change in treatment response based on IVIS imaging (TRT) and weight (delta TRT and delta weight were the outcomes). In these models, pair-wise comparisons were then made between groups for multiple comparisons, with a Bonferroni-adjusted *p*-value being used to determine significance (0.05/28 = 0.00178 (total number of possible pair-wise comparisons)). Thus, all pair-wise comparisons that had *p*-values less than 0.0017 were considered statistically significant. The post hoc comparisons between groups was performed using the ANOVA models so that the variance for the testing was based on all data provided from all eight groups of interest.

## 3. Results

### 3.1. CF10 Displayed Enhanced Potency to CRC Cells Relative to 5-FU and TFT

Previous studies including analysis in the NCI60 cell line screen [14,15] have demonstrated that CF10 (Figure 1; Supplementary Figure S1) displays greatly enhanced potency to CRC cell lines relative to 5-FU, floxuridine, and TFT, significantly exceeding that anticipated based upon a 10-fold greater FP content and more than 100-fold relative to TFT and 5-FU. Further, CF10 was highly potent regardless of MSI/MSS status or KRAS mutation status, consistent with its broad use for CRC treatment regardless of the factors used to stratify CRC patients for 5-FU-based chemotherapy [28]. To gain further insights into the activity of CF10 in cancer cells, we provided a sample to the Broad Institute PRISM screen for testing. Analysis of AUC values for CF10 revealed that relative to other FPs for which PRISM screen data were available, CF10 was highly potent, with AUC values for many CRC cell lines < 0.4, consistent with strong activity (Figure 1C,D). The average AUC value for CF10 in the 14 CRC cell lines tested in the PRISM screen was 0.40, while for 5-FU, the average AUC value was 0.83 (Supplementary Table S1), and for trifluridine, the average AUC value was 0.63 (Supplementary Figure S2, Supplementary Tables S2 and S3). Since previous studies including analysis of NCI60 cell line screen data revealed surprisingly strong mechanistic correlations between F10 and CF10 with DNA topoisomerase 1 poisons, we performed regression analysis of the AUC correlation data from the PRISM screen between FPs and Top1 poisons (Supplementary Figure S3). A strong correlation between camptothecin (CPT) and SN-38 (the active metabolite of irinotecan) was observed (5 × 10^−37^) as expected (Supplementary Table S4). Consistent with previous studies, the correlation between CF10 and Top1 poisons was very strong [14,15], and the AUC correlation for CF10 and SN-38 was very strong (5 × 10^−24^), which was much stronger than that for either TFT or 5-FU with SN-38 (1.1 × 10^−16^ and 1.1 × 10^−4^; Supplementary Table S4). These results are consistent with the increased potency for CF10 resulting from a TS/Top1 dual-targeting mechanism that is more pronounced for CF10 than for other FPs.

### 3.2. FP Potency Was Enhanced by Folate Restriction and LV Co-Treatment

While screens involving large numbers of cell lines are highly valuable in assessing compound potency and identifying genomic determinants of drug response, a limitation of screens such as the NCI60 [13] and the Broad Institute Prism screen [29] is that drug response is evaluated using media that may not reflect human physiology. This is a concern for FP drugs that are highly dependent on folate levels since ATCC-recommended media include much higher folate concentrations than does human plasma. To investigate if the potency advantage for CF10 relative to alternative FPs was realized in cells cultured using media with human-like folate levels, we adapted three human CRC cell lines (HCT116, LS174T, HCT15) and MC38 mouse CRC cells to culture media developed for testing drug activities under conditions of physiological folate [12]. Cells adapted to folate-restricted media appeared healthy (Supplementary Figure S4A) and had growth characteristics similar to those of the same cell line cultured in ATCC-recommended media (Supplementary Figure S4B). However, culture using FR media caused all three CRC cell lines to display increased sensitivity to the three FPs tested (CF10, 5-FU, TFT) (Supplementary Figure S4C).

**Figure 2:**
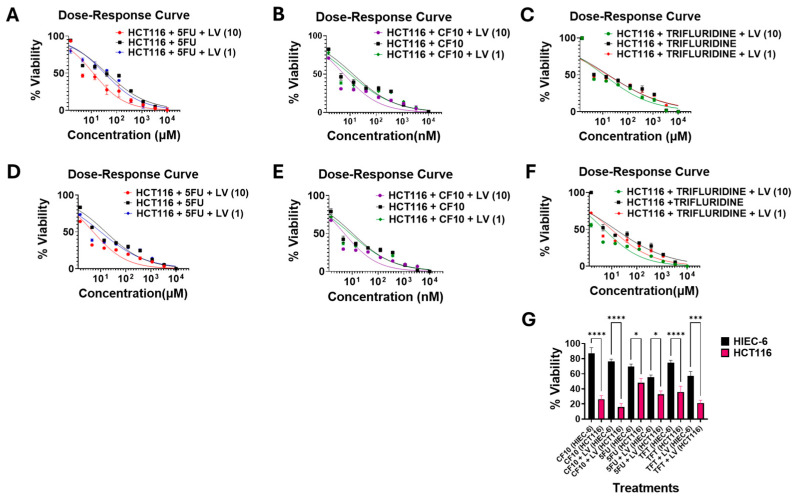
CF10 displayed greatly increased potency to HCT116 cells relative to 5-FU and TFT under standard and folate-restricted cell culture conditions (**A**–**G**). Dose–response curves for (**A**,**D**) 5-FU (black) + LV (1 µM (blue) or 10 µM (red), (**B**,**E**) CF10 (black) + LV (1 µM (green) or 10 uM (purple), and (**C**,**F**) TFT (black) + LV (1 µM (red) or 10 µM (green). (**A**–**C**) HCT116 cells were cultured in complete media. (**D**–**F**) HCT116 cells were cultured in folate-restricted media. (**G**) Graphs of % viability in HCT116 (red) and HIEC-6, a normal intestinal cell line, following 48 h treatment with CF10 (1 µM), 5-FU (10 µM), TFT (10 µM), or LV (1 µM). *p*-values (*n* = 3, * *p* < 0.03; *** *p* < 0.0002; **** *p* < 0.0001).

We then performed dose–response studies evaluating the response of these three CRC cell lines to CF10, 5-FU, and TFT with studies conducted using both complete (DMEM + 10% FBS) and FR media. Results for HCT116 are summarized in Figure 2A–F, and results for LS174T, HCT15, and MC38 are displayed in Supplementary Figures S5–S7A–F and Supplementary Tables S5–S7.

Results for HCT116 relative to the normal intestinal cell line HIEC-6 are shown in Figure 2G. Results for HCT116 cultured in complete media were similar to those reported in previous studies and from the NCI60 cell line screen and were consistent with PRISM screen data (Figure 1B,C). CF10 was by far the most potent of the three FPs tested (IC_50_ = 13.1 nM in HCT116). TFT was approximately 127-fold less potent than was CF10 (IC_50_ = 1.66 µM), and TFT was only slightly more potent (2.46-fold) than was 5-FU (IC_50_ = 4.08 µM) under these culture conditions. CF10 was slightly more potent to HCT116 cells cultured in FR-media (IC_50_ = 10.5 nM) than in complete media, with similar trends and similar relative potencies in FR-media for TFT and 5-FU. All three FPs displayed increased cytotoxicity to HCT116 relative to the normal intestinal cell line HIEC-6; however, the relative differential was greatest for CF10 (Figure 2G).

In clinical regimens for CRC, 5-FU is almost always co-administered with leucovorin (LV). LV potentiates 5-FU cytotoxicity to CRC cells by promoting ternary complex formation involving (i) the active FP metabolite fluorodeoxyuridylate, (ii) the target enzyme thymidylate synthase (TS), and (iii) the reduced folate co-factor N5,N10-tetrahydrofolate, to irreversibly inhibit TS activity and de novo thymidylate biosynthesis (Figure 1B) [1]. We evaluated the effects of LV co-treatment on the cytotoxicity of all three FPs in HCT116 (Figure 2A–F, Table 1), the normal intestinal cell line HIEC-6 (Figure 2G), LS174T (Supplementary Figure S5 and Supplementary Table S5), HCT15 cells (Supplementary Figure S6 and Supplementary Table S6), and MC38 (Supplementary Figure S7 and Supplementary Table S7).

Co-treatment of any of the three FPs with 1 µM of LV resulted in slightly increased potency. Culturing of HCT116 cells in DMEM 5-FU/LV was approximately 1.24-fold more potent than culturing in 5-FU (IC50 = 3.30 µM vs. 4.08 µM), and for HCT116 cells cultured in FR media, the LV effect was slight greater (approximately 1.56-fold, IC50 = 1.01 µM vs. 1.58 µM). Similar trends were found for CF10 and TFT. For HCT116 cells co-treated with 10 µM of LV, the effects were larger, with a 3.40-fold potentiation for 5-FU in DMEM and a 3.12-fold potentiation for 5-FU in FR media. The LV potentiation effects were slightly reduced but still significant for CF10 (2.25, 2.10-fold) and TFT (1.52, 2.85-fold) in DMEM and FR media, respectively. These results are consistent with LV potentiating CF10 as established for 5-FU, but with slightly attenuated effects. Although LV is not presently used in clinical regimens in which TFT is the FP (e.g., TAS-102), LV also potentiates TFT, potentially by affecting nucleotide biosynthesis.

### 3.3. LV Stimulated TS Ternary Complex Formation for CF10, 5-FU but Not TFT

**Figure 3:**
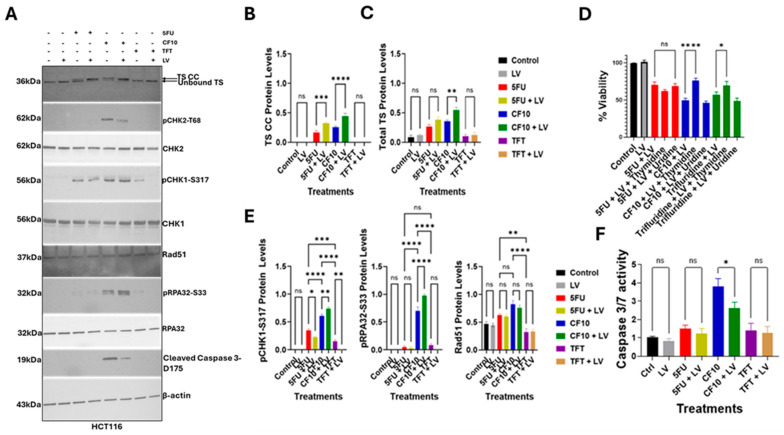
CF10 promoted TS classic complex (TS CC) formation and activated the ATR/Chk1 and ATM/Chk2 DNA damage response pathways in HCT116 cells under folate-restricted culture conditions. (**A**) Western blot for 5-FU ± LV, CF10 ± LV, and TFT ± LV detecting the TS CC and unbound TS, as well as for the protein biomarkers for activation of the ATR/Chk1 (pChk1-S317, pRPA32-S33) and the ATM/Chk2 (pChk2-T68) DNA damage response pathways. Upregulation of the homologous recombination protein Rad 51 involved in DNA double-strand break repair and of cleaved caspase 3 is also shown. The uncropped blots are shown in File S1. (**B**,**C**) Quantification of TS CC and total TS levels. (**D**) Effect of thymidine (80 µMol) and uridine (1 mMol) co-treatment on HCT-116 cell viability in FR media for 5-FU + LV (red), CF10 + LV (blue), and TFT + LV (green). FP + LV was dosed at IC_50_ for 48 h, as shown in Figure 2. Experiments were performed in triplicate ± SEM (* *p* < 0.03; ** *p* < 0.002; *** *p* < 0.0002; **** *p* < 0.0001; ns—not significant). Decreased cell viability for CF10 + LV and TFT + LV was partly reversed by thymidine co-treatment but not for 5-FU + LV. (**E**) Quantification of A for pChk1-S317, pRPA32-S33, and Rad 51 levels from densitometry of A. (**F**) Caspase 3/7 Glo assay results showing CF10 induces apoptosis in HCT116 cells while CF10 + LV induces less apoptosis.

To determine if LV co-treatment with 5-FU, CF10, and TFT stimulated TS ternary complex formation (FdUMP/TS/5,10-methylene THF), we performed Western blots using an anti-TS antibody under conditions that resolved the ternary complex (aka TS classic complex (TS CC) [30]) and the unbound enzyme. Results in HCT116 cells are displayed in Figure 3A, and the quantification of TS CC and total TS is displayed in Figure 3B,C.

The results in LS174T, HCT15, and MC38 cells are shown in Supplementary Figures S8–S10. In the absence of any FP treatment, TS CC was not detected. However, TS CC was detected as a minor component with 5-FU treatment, and with CF10, TS CC was the predominant form present. Interestingly, total TS levels were reduced with CF10 treatment relative to no treatment (Figure 3B,C), which is consistent with CF10 stimulating increased TS degradation [31]. While TFT is a substrate for thymidine kinase and known to inhibit TS, TFT treatment did not result in detectable TS CC either as a single drug or with TFT + LV co-treatment (Figure 3A). Consistent with clinical objectives, LV co-treatment with 5-FU did increase TS CC levels and in contrast to observed effects of CF10 in decreasing TS intensity with LV co-treatment, 5-FU intensified the TS CC band, which was the predominant form (Figure 3B,C), a result consistent with 5-FU potentially stabilizing TS from proteasomal degradation [31]. Consistent with results showing CF10 and CF10/LV were effective at increasing TS CC formation and reducing TS levels, TS enzymatic activity was strongly reduced with these treatments (Supplementary Figure S11).

To determine if LV co-treatment altered the effects of a thymineless state in promoting FP cytotoxicity, we performed reversal studies in which added thymidine or uridine were included in the culture medium during treatment. Results for HCT116 cells are displayed in Figure 3D, and results for LS174T, HCT15, and MC38 cells are displayed in Supplementary Figures S8–S10. 5-FU cytotoxicity can occur through either an RNA- or DNA-directed mechanism, depending on the cell type. While TS CC was evident as the predominant TS form in HCT116 cells with 5-FU/LV co-treatment, cytotoxicity was not reversed with either Thy or Urd. Consistent with CF10 cytotoxicity resulting from TS inhibition, co-treatment with Thy reduced CF10 cytotoxicity, while Urd co-treatment had no effect. Thy co-treatment also reversed TFT cytotoxicity despite the absence of detectable TS CC, but effects were attenuated relative to CF10. Similar effects were detected in HCT15, LS174T, and MC38 cells (Supplementary Figures S8–S10).

### 3.4. CF10 Was a Potent Inducer of Replication Stress and Apoptosis

In previous studies, we established that CF10 enhanced replication stress in CRC cells as shown by the increased stalling and collapse of replication forks [6]. CF10 induces replication stress through the dual targeting of TS and Top1 [6,18], which results in trapped Top1 cleavage complexes (Top1cc) [32,33,34] that impede replication fork progression while reducing thymidine levels due to TS inhibition hinder DNA repair. Top1cc is converted to DNA double-strand breaks (DSBs) upon collision with advancing replication forks or during transcription [35], and this contributes to genomic instability and can activate programmed cell death. In these studies, we showed that CF10 is much more potent than a 5-FU or TFT to CRC cells (Figure 2A–F, Table 1), including culture under conditions of folate restriction (Table 1). To determine if CF10 enhances markers of replication stress in CRC cells cultured in folate-restricted media and if LV co-treatment affects DNA damage checkpoint activation, we performed Western blots for markers of DNA damage response (DDR) checkpoint activation, with results for HCT116 cells shown in Figure 3E and the results for LS174T, HCT15, and MC38 cells shown in Supplementary Figures S8–S10. Replication fork stalling activates the ATR/Chk1 pathway and enables the intra-S-phase DDR checkpoint, which inhibits the initiation of new replication forks and provides time to re-start stalled replication forks and repair DNA DSBs. CF10 treatment significantly increased pChk1 Serine-317 (pS317) levels and pRPA32 Serine-33 (pS33) consistent with ATR/Chk1 pathway activation. Treatment with 5-FU also activated the ATR/Chk1 pathway; however, quantification of Western blot intensity by densitometry showed significantly reduced intensity for pChk1 (S317) and pRPA32 (S33) for 5-FU relative to CF10. For TFT, the intensities for these bands were reduced further (Figure 3E). Since Top1cc formation can cause replication fork stalling and activation of the ATR/Chk1 pathway, we evaluated Top1cc and showed increased levels in HCT116 cells with CF10 and CF10/LV relative to other treatments (Supplementary Figure S12).

The ATM/Chk2 pathway was also selectively activated with CF10 treatment in HCT116 cells cultured in FR media relative to 5-FU and TFT as shown by Western blot for pChk2-threonine68 (T68) (Figure 3E). Immunofluorescence for gH2AX was also greatest for CF10 relative to other FPs (Supplementary Figure S12B). Consistent with generation of DNA DSBs with CF10 treatment, Rad51 levels were increased but were not increased in CRC cells treated with either 5-FU or TFT. To determine if LV co-treatment affected the activation of the ATR/Chk1 or ATM/Chk2 pathways, we also analyzed lysates from CRC cells with FP + LV co-treatment for all three FPs tested (CF10, 5-FU, TFT). LV co-treatment enhanced pRPA32-S33 and pChk1-S317 levels in CF10-treated cells consistent with increased ATR/Chk1 pathway activation. However, levels of pChk2-T68 and Rad51 were decreased with CF10 + LV treatment relative to CF10-only, which is consistent with reduced DNA DSBs.

The extent of apoptosis in FP-treated CRC cells was assessed by Western blot for cleaved caspase 3 (Figure 3E) and caspase 3/7-Glo assay (Figure 3F). Cleaved caspase 3 was not detected with either 5-FU or TFT treatment in HCT-116 cells with or without LV co-treatment (Figure 3E) or in LS174T, HCT15, or MC38 cells, as shown in Supplementary Figures S8–S10. Cleaved caspase 3 was detected with CF10 treatment, and CF10 also induced apoptosis relative to other treatments based on caspase 3/7-Glo. Surprisingly, although LV co-treatment with CF10 decreased cell viability relative to single-agent CF10, the extent of apoptosis induced by CF10 + LV relative to CF10 was less (Figure 3E,F). Our results are consistent with the increased potency of CF10 relative to 5-FU and TFT in human and mouse CRC cells resulting from increased replication stress with ATR/Chk1 and ATM/Chk2 DNA damage response pathways activated to counter the deleterious consequences of CF10-induced Top1cc and replication fork collapse resulting in apoptosis, although the non-apoptotic cell death processes may be stimulated by CF10/LV since increased potency correlates with reduced apoptosis.

### 3.5. CF10 Was Highly Effective at Eradicating Liver-Metastatic Tumor Burden

**Figure 4:**
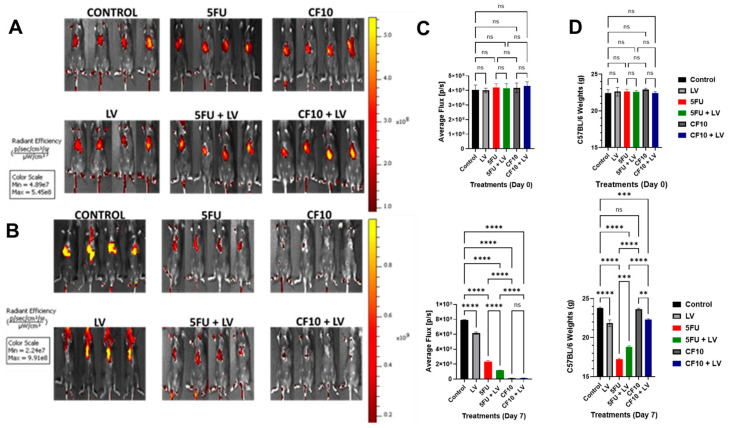
CF10/LV was more effective than 5-FU/LV in a liver-metastatic CRC model under conditions of human-like folate levels. (**A**,**B**) IVIS images of tumor burden for indicated treatments at Day 0 (**A**) and Day 7 (**B**) post-treatment. (**C**) Tumor flux pre-treatment (**top**) and post-treatment (**bottom**). (**D**) Mice weights pre-treatment (**top**) and post-treatment (**bottom**). *p*-values (*n* = 4, ** *p* < 0.002; *** *p* < 0.0002; **** *p* < 0.0001; ns—not significant).

In previous studies, we established that CF10 displays more potent antitumor activity than did 5-FU in a mouse orthotopic model of primary colon cancer (HCT116/Balbc nu/nu) [6] and in a rat CRLM model (CC531/WAGRij) [7]. In the latter study, rats were injected 1×/wk with 5-FU or CF10. To better emulate clinical dosing with 5-FU, which is usually done with infusion or bolus + infusion as in FOLFOX6 [36], we evaluated the antitumor activity of 5-FU or CF10 infused over 7 days using an osmotic pump. Further, mice were adapted to a folate-restricted diet to study tumor progression and treatment response under more human-like folate levels. We compared the antitumor activity of 5-FU and CF10 with and without LV since 5-FU is generally used with LV for CRC, and our studies demonstrated that LV co-treatment potentiates CF10 (Table 1). TFT, which is used to treat third-line mCRC as part of TAS-102 was also tested in these studies. TFT was administered as a single agent (TAS-102 includes the thymidine phosphorylase inhibitor) and was infused over 7 days, identical to the schedule used for 5-FU and CF10 in these studies. All treatment groups received equivalent amounts of FP based on UV activity to account for the 10-fold higher FP content of CF10 on a molar basis. The results following 7-day infusion for 5-FU ± LV and CF10 ± LV are shown in Figure 4 while those for TFT ± LV are shown in Supplementary Figure S13.

Liver-metastatic tumor burden was initiated by injecting MC38 cells into one lobe in the liver of C57Bl/6 mice. Seven days prior to tumor cell injection, mice were adapted to a folate-restricted diet to develop human-like folate physiology which is important for evaluating the activity of FP drugs in rodents [37]. Seven days’ post-injection, tumor formation was validated in mice by fluorescence imaging using IVIS following injection of a fluorescent RGD peptide. Initial tumor levels were similar in all mice, and tumor was localized to the liver (Figure 4A). Mice were treated with a 7-day infusion of one of the FPs (5-FU, CF10, TFT) via an Alzet mini-pump fitted with a jugular vein catheter. 5-FU was administered at 100 mg/kg, and doses of CF10 and TFT were matched to the 5-FU dose based on equal A260 absorbance. Groups of *n* = 4 mice received (i) no treatment, (ii) single-agent 5-FU, (iii) single-agent CF10, (iv) single-agent TFT, (v) single-agent LV, (vi) 5-FU + LV, (vii) CF10 + LV, or (viii) TFT + LV. The effects of treatment on tumor progression were evaluated by IVIS imaging following completion of the 7-day infusion (Figure 4B). All treatment groups, including single-agent LV, displayed reduced flux values consistent with tumor reduction (Figure 4C). Among the three FP treatment groups, the flux decrease was least for single-agent 5-FU, and while this decreased with LV co-treatment, flux reduction with 5-FU + LV treatment was still not as large as that with CF10 or TFT, either as single agents or in combination with LV. Substantial flux reduction was observed with CF10 and TFT treatment as single agents and in combination with LV (Figure 4C) (Supplementary Table S8).

Mice in all groups tolerated treatment; however, weight loss was observed for all treatment groups except for single-agent CF10, for which mice gained weight during treatment. Weight loss was significant for 5-FU, 5-FU + LV, TFT, and TFT + LV (Supplementary Table S9). The largest weight loss occurred with 5-FU, and despite mice treated with single-agent LV losing weight, mice treated with 5-FU + LV lost less weight than did those treated with single-agent 5-FU. Weight loss for mice treated with CF10 + LV was slightly less than that for mice treated with single-agent LV. Mice treated with single-agent TFT underwent significant weight loss but not as great as that in those treated with 5-FU, and combining TFT + LV did not increase weight loss (Supplementary Table S9).

**Figure 5:**
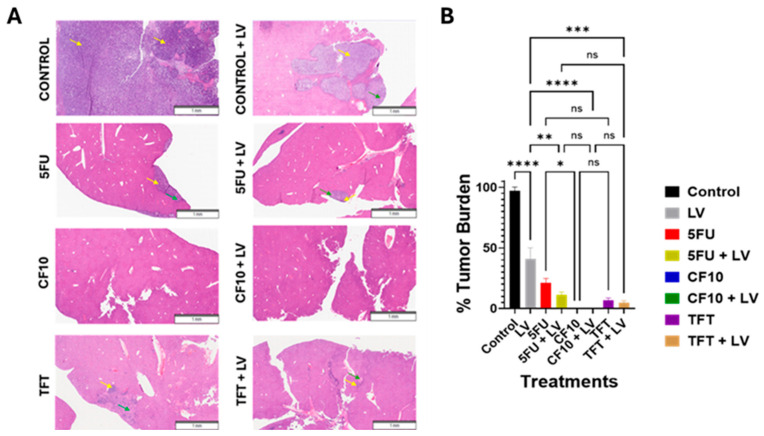
Pathological review of liver H&E sections from the mouse liver metastasis study demonstrated that CF10 ± LV eradicates liver metastases without scarring. (**A**) H&E-stained liver sections from mice with the indicated treatments (yellow arrows point to residual tumor and green arrows to scarring/inflammation). (**B**) Quantification of residual tumor from (**A**). *p*-values (*n* = 4, * *p* < 0.03; ** *p* < 0.002; *** *p* < 0.0002; **** *p* < 0.0001; ns—not significant).

To gain further insight into the effects of treatment on tumor progression, mice were euthanized 7 days after treatment following IVIS imaging, and livers were excised. H&E-stained sections from livers of all treatment groups were analyzed by a pathologist to evaluate tumor progression (Figure 5A).

Extensive tumor was found in livers from all control mice. However, all treatment groups, including single-agent LV, displayed reduced tumor burden relative to control (Supplementary Table S10). Treatment with 5-FU and 5-FU/LV was less effective relative to that with CF10 and TFT. CF10 as a single agent and in combination with LV completely eradicated tumor, with no early scarring or inflammation noted upon pathological review (Figure 5A,B). TFT was effective in this tumor model; however, inflammation and early scar formation were noted (Figure 5A, Supplementary Table S10), and unlike for CF10 and CF10/LV, residual tumor was evident (Figure 5A, Supplementary Table S10). These results are consistent with the established clinical efficacy for 5-FU/LV and TFT (as part of tiperacil) in CRLM treatment and indicate CF10 as single agent or as CF10 + LV is likely to be highly active in treating liver-metastatic burden.

## 4. Discussion

Fluoropyrimidine (FP) drugs are central to disease management for metastatic CRC patients, and treatment with FP-based regimens provides proven survival benefits. Many mCRC patients survive to receive three or more lines of chemotherapy, with each including an FP drug. The 5-FU/LV-based regimens FOLFOX and FOLFIRI are widely used in first- and second-line chemotherapy for mCRC, while TAS-102 that combines the FP TFT with the thymidine phosphorylase (TP) inhibitor tiperacil is approved for third-line mCRC treatment for patients previously treated with 5-FU/LV and other drugs [2]. While mCRC patients are increasingly living longer due to optimized drug scheduling and sequential order, long-term survival remains rare, with only <14% of mCRC patients surviving five years’ post-diagnosis. In this study, we highlighted the improved activity of a nanoscale DNA-based FP polymer, CF10, relative to that achieved by the legacy FP drugs widely used in mCRC treatment, 5-FU and TFT. Importantly, we established a therapeutic benefit for CF10 relative to 5-FU with infusion dosing, demonstrating that the therapeutic advantages of CF10 cannot be achieved with 5-FU simply through schedule optimization.

In previous testing in our laboratory [6] and in the NCI60 cell line screen [14], CF10 and F10 displayed potency advantages substantially greater than those anticipated based upon a 10-fold greater FP content exceeding 1000-fold relative to 5-FU in some cancer cell lines [15]. In these studies, we extended these findings in the Broad Institute PRISM screen. Analysis of AUC value, an indicator of drug potency, with strong potency correlated with a low AUC, revealed much lower values for CF10 relative to 5-FU and TFT, consistent with increased potency broadly across all CRC cell lines tested (Figure 1C,D; Supplementary Tables S1–S3). While results from large cell screens can be extremely valuable and potentially enable dose–response data to be correlated with genomic and proteomic factors, screen data must be interpreted with caution since cell–compound interactions that may be important for predicting in vivo efficacy may not be represented ideally under the screening conditions. One factor identified as important for FP cytotoxicity is folate levels in culture media [11]. In these studies, we tested the relative potency of CF10, 5-FU, and TFT in both complete media and in media with restricted folate (FR media) to better simulate human physiology. The potency advantage for CF10 relative to 5-FU and TFT was retained to CRC cells in FR conditions (Figure 2A–F and Table 1) without causing increased cytotoxicity to non-malignant cells (Figure 2G). A potentiating effect of LV co-treatment was also evident for CF10 in CRC cells cultured under FR conditions and was similar as that for 5-FU (2.10- vs. 3.12-fold for HCT-116) (Figure 2A–F). The data support LV co-treatment being beneficial during CF10 clinical development. TFT potency was also increased by LV co-treatment, and although TAS-102 is not combined with LV, our studies indicate that doing so could benefit CRC patients.

The cytotoxic mechanism responsible for CF10′s enhanced potency to CRC cells involves dual targeting of TS and Top1. The effects of CF10 on these enzymes are mediated through three metabolites, FdUMP, FdUTP, and AraCTP. Nuclease degradation of CF10 releases FdUMP without the multiple steps of anabolic metabolism required for the inefficient conversion of 5-FU to FdUMP [38]. Western blots of TS from FP-treated cells cultured under FR conditions showed that with CF10, but not 5-FU treatment, TS was predominantly complexed with FdUMP and migrated as a band with slightly reduced intensity (TS CC; Figure 3A), a result consistent with higher FdUMP levels in CF10-treated cells. FdUMP not complexed with TS may be metabolized to FdUTP, and under the thymineless conditions caused by TS inhibition, FdUTP is incorporated into DNA during replication in place of thymidine. We previously established that FdU interferes with the re-ligation step of Top1 catalysis [14], resulting in the formation of a DNA–protein complex (i.e., Top1 cleavage complex (Top1cc). Similarly, AraCTP misincorporation into DNA results in Top1cc formation by a similar mechanism [16]. Top1cc formed with either nucleoside analogs or classic Top1 poisons (e.g., camptothecin) is converted to DNA double-strand breaks (DSBs) by collision with either an advancing replication fork or during transcription of the DNA containing the Top1cc [32,33,34]. Our data show that CF10/LV causes formation of Top1cc as evidenced from immunofluorescence (Supplementary Figure S12). Analysis of correlations from PRISM screen data for FPs (CF10, 5-FU, TFT) and SN-38 (the active metabolite of irinotecan—Supplementary Figure S3) showed that CF10 had much stronger correlation with SN-38 (5 × 10^−24^) than did TFT (1.1 × 10^−16^) or 5-FU (1.1 × 10^−4^) (Supplementary Table S4). These results are consistent with CF10 inducing enhanced replication stress (Figure 3E,F) resulting from TS/Top1 dual targeting. This dual targeting contributes to CF10′s increased potency of CF10 relative to clinically used FPs. Our data demonstrate this mechanism occurs for CF10/LV and under folate-restricted conditions.

The present studies also addressed whether CF10 could be more effective than 5-FU in the treatment of CRC liver metastasis [39] under conditions of physiological folate. In clinical regimens, 5-FU may be administered by bolus injection, infusion, or both, with infusion often performed over ~46 h [40]. CF10 is a DNA-based FP polymer, and while clinical data are not available for CF10, there is clinical precedent for oligonucleotide activity with long infusions, such as those lasting 21 days [41]. In the present study, we tested CF10 and 5-FU with delivery of equivalent FP levels based on A260 absorbance and with infusion over seven days using an osmotic mini-pump fitted with a jugular vein catheter. We elected to test TFT under identical conditions. Thus, while TFT is orally administered as part of TAS-102 [42], we delivered the equivalent amount based on fluoropyrimidine monomer via the same route to enable direct comparison of in vivo antitumor activity under similar conditions. Liver tumor masses were formed by the injection of MC38 mouse CRC cells into one lobe of the liver in syngeneic host C57BL/6 mice [43], similar to our previous study using a syngeneic rat model of orthotopic CRC liver metastasis [7,21]. Mice have considerably higher folate levels than do humans, and mice were fed a diet shown previously to reduce folate levels to more human-like levels [44,45,46]. 5-FU and 5-FU/LV reduced tumor progression in this CRC liver metastasis model, consistent with the clinical efficacy of 5-FU/LV in mCRC treatment [47]. Consistent with previous studies in which we evaluated CF10 and 5-FU in primary colon cancer [6] and in a rat CRC liver metastasis model [7], CF10 was more effective than 5-FU. These results extend previous findings in several ways that are important for CF10 translation. The improved activity of CF10 relative to 5-FU was achieved in the context of relatively lower folate levels, more similar to human physiology. The strong antitumor activity of CF10 was also achieved without weight loss, while equivalent amounts of both 5-FU and TFT caused significant weight loss in treated mice. CF10 also caused no scarring of the liver, which was evident with both 5-FU and TFT. Importantly, CF10 completely eradicated the liver tumor mass in this liver metastatic model, which neither 5-FU ± LV nor TFT was able to do. Since infusion dosing was used for all treatments in this study and previous studies have shown increased efficacy for CF10 relative to 5-FU with bolus dosing, our study demonstrated an improved therapeutic benefit for CF10 regardless of schedule. CF10/LV displayed equivalent antitumor activity to that of single-agent CF10 without weight loss or liver scarring, and based on the enhanced potency in cell-based assays, the data support the clinical development of CF10/LV.

## 5. Conclusions

Overall, our findings demonstrate that CF10 is much more potent in CRC cells than are FPs used in treatment regimens for mCRC. The increased potency of CF10 relative to 5-FU and TFT is general—as evidenced by lower AUC values over multiple CRC cell lines included in the Broad Institute PRISM screen is maintained with folate restriction, and is associated with stronger TS inhibition and potent Top1cc formation, consistent with mechanistic similarities to Top1 poisons [48]. LV co-treatment [49] enhances CF10 cytotoxicity to CRC cells, and while the effect is less pronounced than is that for 5-FU, our results support co-treatment with LV or alternative reduced folate [50] during CF10 development. The finding of complete eradication of tumor mass in a CRC liver metastasis model with CF10 infusion but of 5-FU/LV infusion only inducing tumor regression but not eradication is encouraging for the clinical development of CF10. TFT was highly effective in this model, consistent with clinical activity, but treatment-related scarring was detected with TFT, which also caused weight loss. Our findings support advanced pre-clinical testing of CF10 and the initiation of clinical studies with strong potential for CF10 to contribute to long-term survival in mCRC patients.

## Figures and Tables

**Table 1:** Summary of IC_50_ values and LV potentiation effects for HCT116 cells.

FR Drug	Media	IC_50_ (µM) ± SEM	IC_50_ (µM) + LV (1 µM) ± SEM	IC_50_ (µM) + LV (10 µM) ± SEM	LV Effect (1 µM)	LV Effect (10 µM)
5FU	DMEM	4.08 ± 0.0133	3.30 ± 0.0151	1.20 ± 0.0122	1.24	3.40
5FU	FR	1.58 ± 0.0177	1.01 ± 0.0136	0.506 ± 0.0157	1.56	3.12
CF10	DMEM	0.0131 ± 0.000125	0.0101 ± 0.000132	0.00583 ± 0.000138	1.30	2.25
CF10	FR	0.0105 ± 0.000132	0.00842 ± 0.000145	0.00501 ± 0.000144	1.25	2.10
TFT	DMEM	1.66 ± 0.0105	1.54 ± 0.0105	1.09 ± 0.0112	1.08	1.52
TFT	FR	1.41 ± 0.0114	0.967 ± 0.0146	0.495 ± 0.0150	1.46	2.85

## Data Availability

The original contributions presented in this study are included in the article. Further inquiries can be directed to the corresponding author.

## Supplementary Materials

The following supporting information can be downloaded at https://www.mdpi.com/article/10.3390/cancers17172739/s1: Figure S1: Structure of CF10; Figure S2: AUC values for trifluorthymidine and 5-FU from the PRISM screen; Figure S3: Plots of AUC for SN-38 vs. (A) 5-FU, (B) CF10, and C TFT; Figure S4: Comparison of cell viability in ATCC-recommended media (CM) and media with human-like folate levels (FR); Figure S5: Summary of GI50 values for LS174T CRC cell line for CF10, 5FU, TFT, and ± LV combination; Figure S6: Summary of GI50 values for HCT15 CRC cell line for CF10, 5FU, TFT, and ± LV combination; Figure S7: Summary of GI50 values for MC38 CRC cell line for CF10, 5FU, TFT, and ± LV combination; Figure S8: CF10 promotes TS classic complex (TS CC) formation and activates the ATR/Chk1 and ATM/Chk2 DNA damage response pathways in LS174T cells under folate-restricted culture conditions; Figure S9: CF10 promoted TS classic complex (TS CC) formation and activates the ATR/Chk1 and ATM/Chk2 DNA damage response pathways in HCT15 cells under folate-restricted culture conditions; Figure S10: CF10 promoted TS classic complex (TS CC) formation and activated the ATR/Chk1 and ATM/Chk2 DNA damage response pathways in MC38 cells under folate-restricted culture conditions; Figure S11: CF10 and CF10/LV were effective at reducing TS enzymatic activity; Figure S12: CF10 and CF10/LV induced Top1cc in HCT-116 cells; Figure S13: TFT and TFT/LV were effective in a liver-metastatic CRC model under conditions of human-like folate levels; Table S1: AUC values for CF10 and 5-FU from the PRISM screen; Table S2: AUC values for CF10 and trifluorothymidine from the PRISM screen; Table S3: AUC values for trifluorothymidine and 5-FU from the PRISM screen; Table S4: Summary of p-values for the correlations of SN-38 with indicated drugs from the Broad Institute PRISM screen; Table S5: Summary of GI50 values for LS174T CRC cell line for CF10, 5FU, TFT, and ±LV combination; Table S6: Summary of GI50 values for HCT15 CRC cell line for CF10, 5FU, TFT, and ±LV combination; Table S7: Summary of GI50 values for MC38 CRC cell line for CF10, 5FU, TFT, and ±LV combination; Table S8: Least squares means analysis of change in IVIS flux intensities during treatment; Table S9: Least squares means analysis of change in weight during treatment; Table S10: Summary of pathological review of liver scarring/inflammation and % tumor burden; File S1: Full pictures of the Western blots in Figure 3A; File S2: Full pictures of the Western blots in Supplementary Figures.
