# Supplementary material for: CF10 Displayed Improved Activity Relative to 5-FU in a Mouse CRLM Model Under Conditions of Physiological Folate
Source: Cancers (Basel). 2025 Aug 23;17(17):2739. doi: 10.3390/cancers17172739 (PMC12427396; doi:10.3390/cancers17172739)
Supplement: Supplementary file 1 [file cancers-17-02739-s001.zip › cancers-3743016 - Supplementary information.pdf]

Supplementary information:

CF10 Displays Improved Activity Relative to 5-FU in a Mouse CRLM Model Under Conditions of Physiological Folate

Charles Chidi Okechukwu[1], Xue Ma[2], Wencheng Li[3], Ralph D'Agostino[4], Jr., Matthew G. Rees[5], Melissa M. Ronan[5], Jennifer A. Roth[5], and William H. Gmeiner[1*]

[1]Department of Cancer Biology, Wake Forest University School of Medicine, Winston-Salem, NC 27157 USA;
[2]Department of Orthopedic Surgery. Wake Forest University School of Medicine, Winston-Salem, NC 27157 USA;
[3]Department of Pathology, Wake Forest University School of Medicine, Winston-Salem, NC 27157 USA;
[4]Department of Public Health Sciences and Comprehensive Cancer Center, Wake Forest University School of Medicine, Winston-Salem, NC 27157 USA;
[5]Broad Institute of MIT and Harvard, Cambridge, MA 02142

Running Title: Improved efficacy of CF10 in liver-metastatic CRC

A

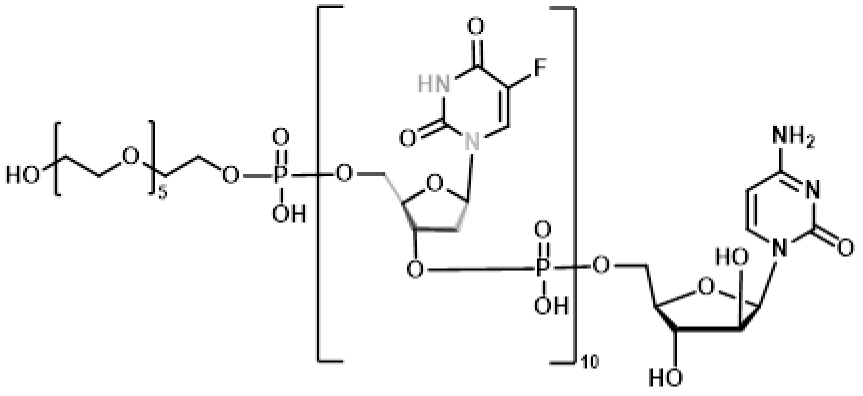

Supplementary Figure S1. Structure of CF10.

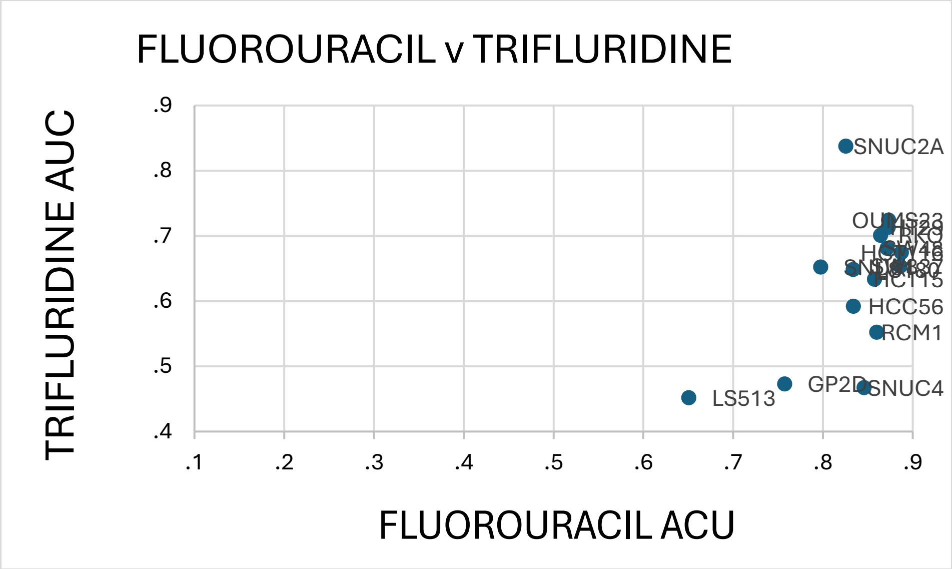

Supplementary Figure S2. AUC values for Trifluorthymidine and 5-FU from the PRISM screen.

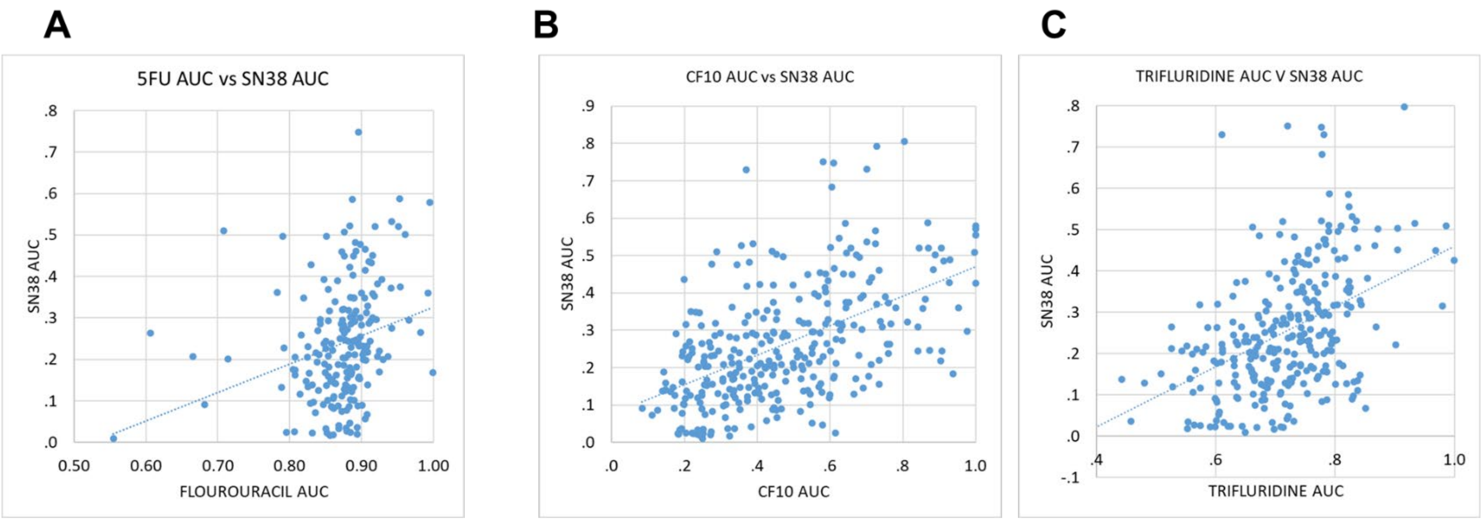

Supplementary Figure S3. Plots of AUC for SN-38 vs (A) 5-FU, (B) CF10, and C TFT. P-values are summarized in Supplementary Table S4.

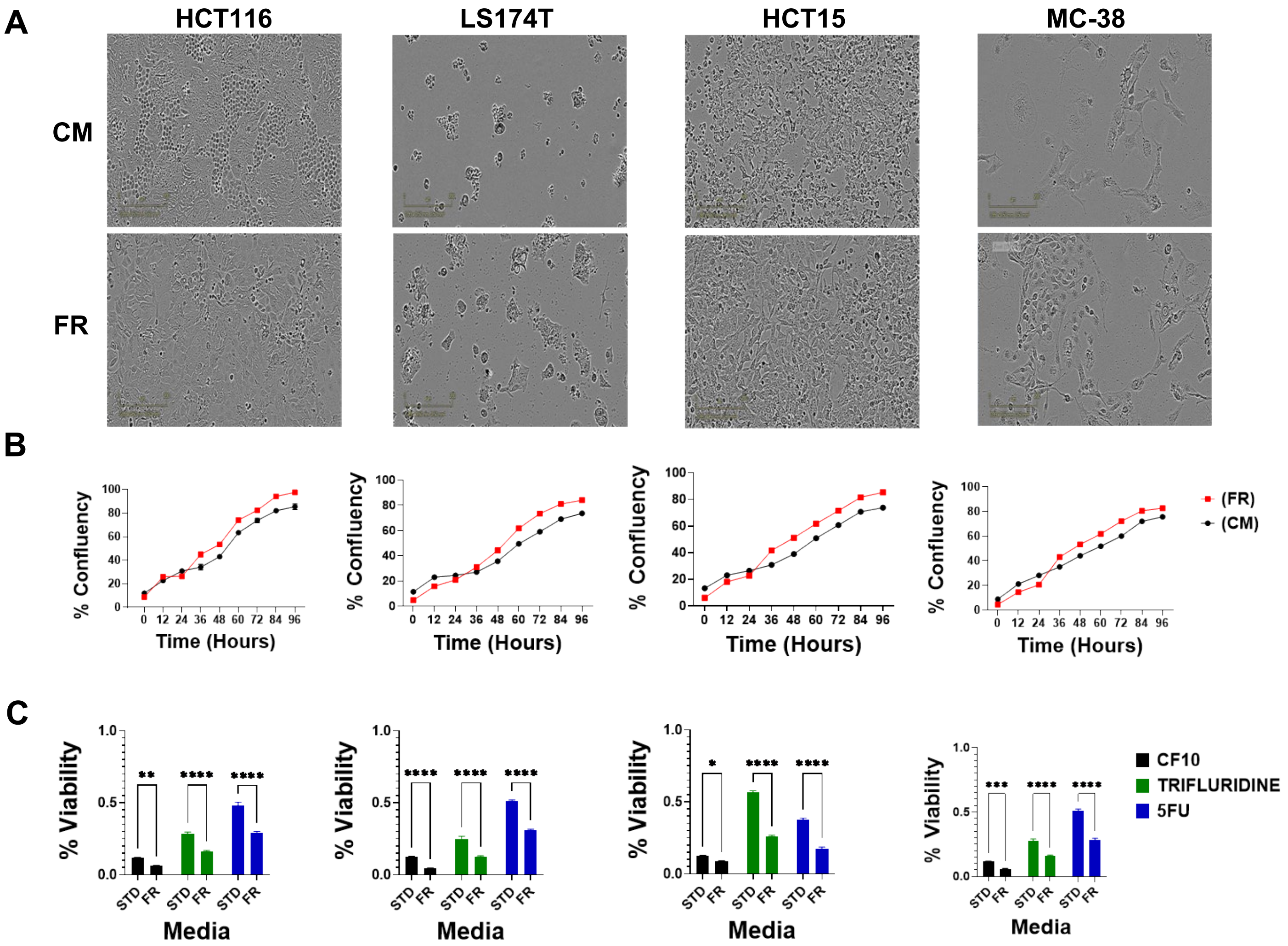

Supplementary Figure S4. Comparison of cell viability in ATCC-recommended media (CM) and media with human-like folate levels (FR). (A) Photomicrographs of cells showing similar morphology and viability in CM and FR media; (B) % confluency in 96 well plates as a function of time showing similar growth rates in FR and ATCC-recommended media; (C) % viability for the indicated cell line in response to treatment with each of the three FPs (CF10, TFT, 5-FU) in ATCC-recommended and FR media.

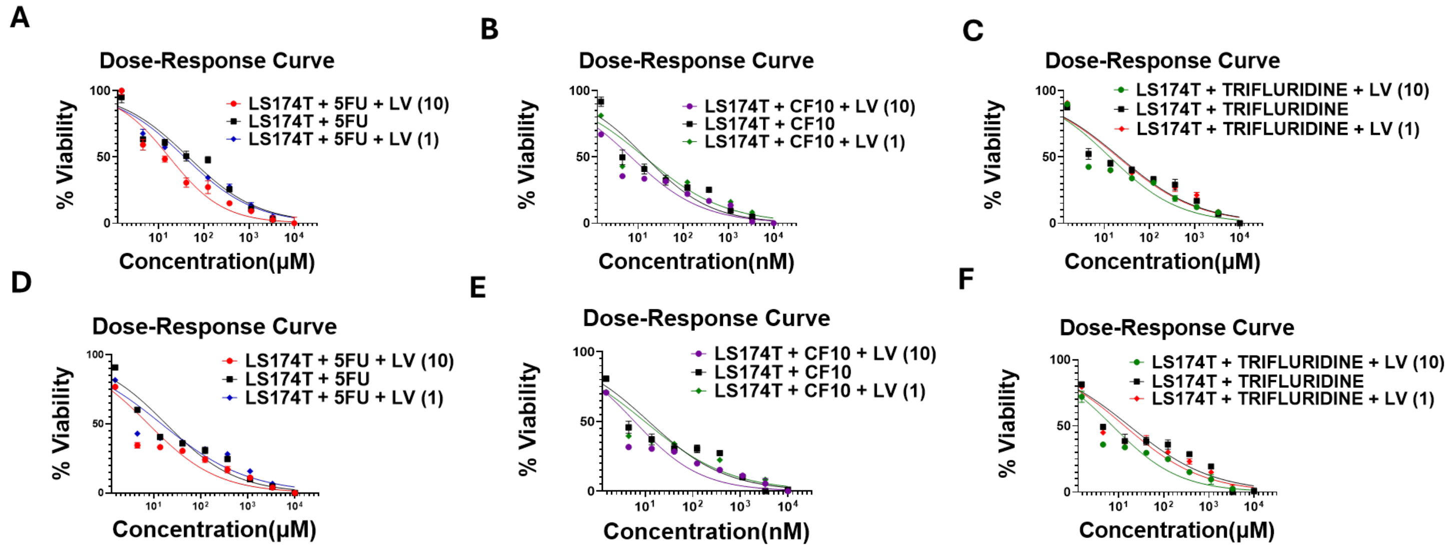

Supplementary Figure S5. Summary of GI50 values for LS174T CRC cell line for CF10, 5FU, TFT, and ± LV combination. CF10 is much more potent to CRC cell than 5-FU and TFT on both standard media and folate-restricted media. LV potentiates the cytotoxicity of CF10 to a similar extent as 5-FU.

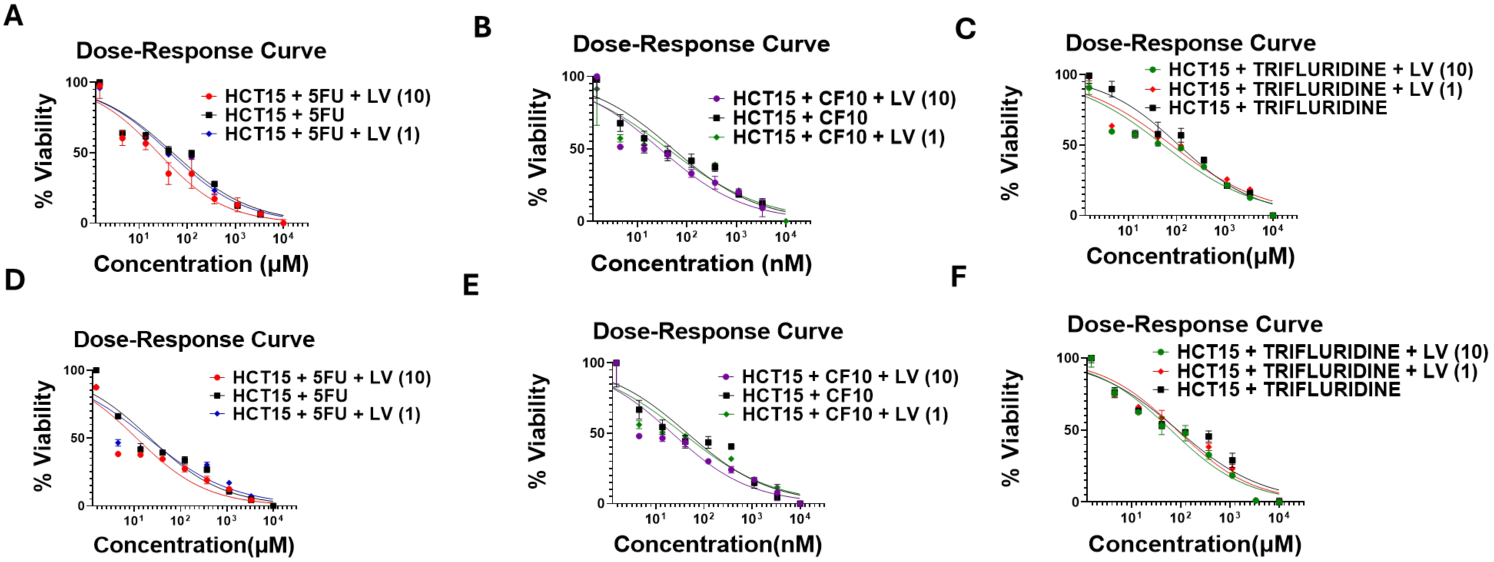

Supplementary Figure S6. Summary of GI50 values for HCT15 CRC cell line for CF10, 5FU, TFT, and ± LV combination. CF10 is much more potent to CRC cell than 5-FU and TFT on both standard media and folate-restricted media. LV potentiates the cytotoxicity of CF10 t

to a similar extent as 5-FU.

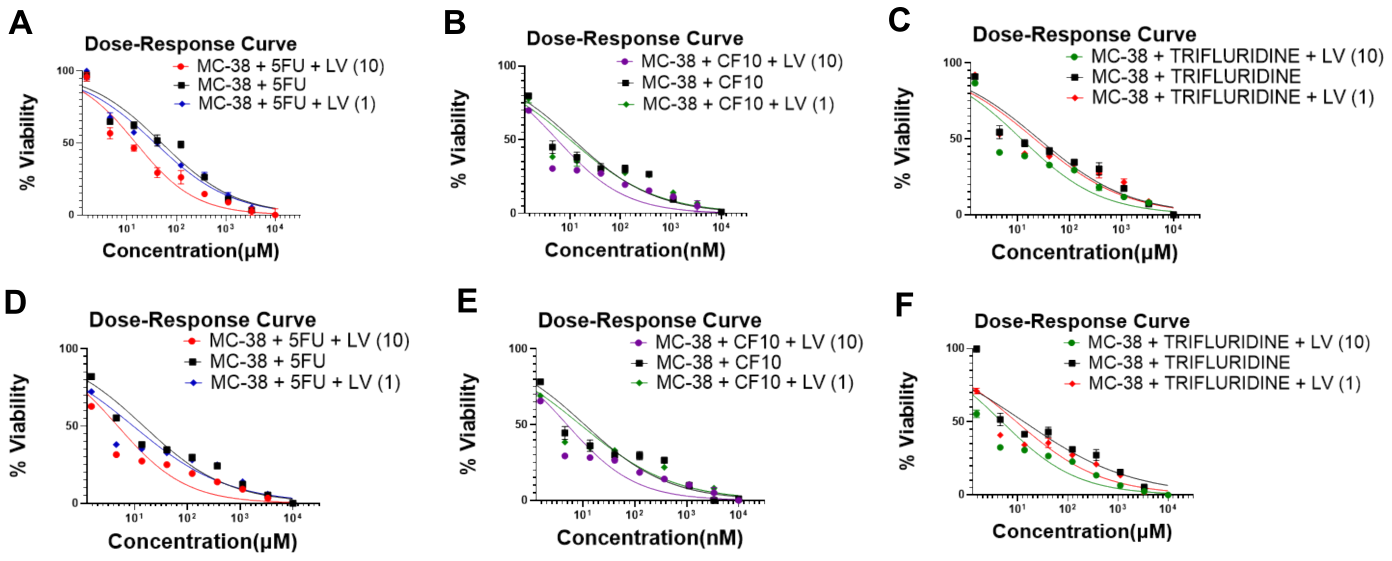

Supplementary Figure S7. Summary of GI50 values for MC38 CRC cell line for CF10, 5FU, TFT, and ± LV combination. CF10 is much more potent to CRC cell than FP drugs.

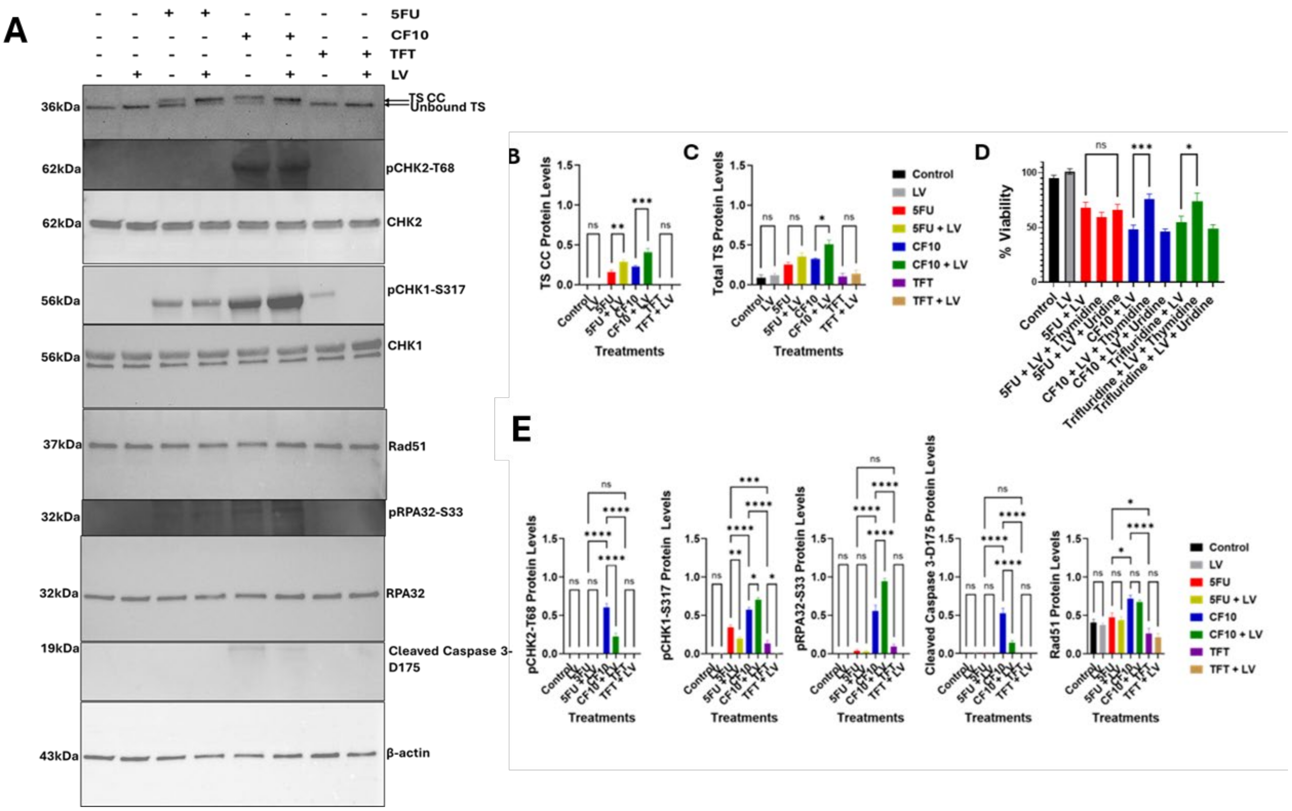

Supplementary Figure S8. CF10 promotes TS classic complex (TS CC) formation and activates the ATR/Chk1 and ATM/Chk2 DNA damage response pathways in LS174T cells under folate-restricted culture conditions. (**A**) Western blot for 5-FU ± LV, CF10 ± LV, and TFT ± LV detecting the TS CC and unbound TS also detecting protein biomarkers for activation of the ATR/Chk1 (pChk1-S317, pRPA32-S33) and the ATM/Chk2 (pChk2-T68) DNA damage response pathways. Upregulation of the homologous recombination protein Rad 51 involved in DNA double strand break repair and of cleaved caspase 3 is also shown; (**B,C**) Quantification of TS CC and total TS levels; (**D**) effect of thymidine (80 uMol) and uridine (1 mMol) co-treatment on HCT-116 cell viability in FR media for 5-FU+LV (red), CF10+LV (blue), and TFT+LV (green). FP+LV was dosed at IC50 for 48h shown in Figure 2. Experiments done in triplicate ± SEM (***p ≤ 0.0002, **p ≤0.002, *p ≤ 0.03). Decreased cell viability for CF10+LV and TFT+LV is partly reversed by thymidine co-treatment but not for 5-FU+LV. (**E**) quantification of (**A)** for pChk1-S317; pRPA32-S33; Rad 51 levels from densitometry of **(A)**.

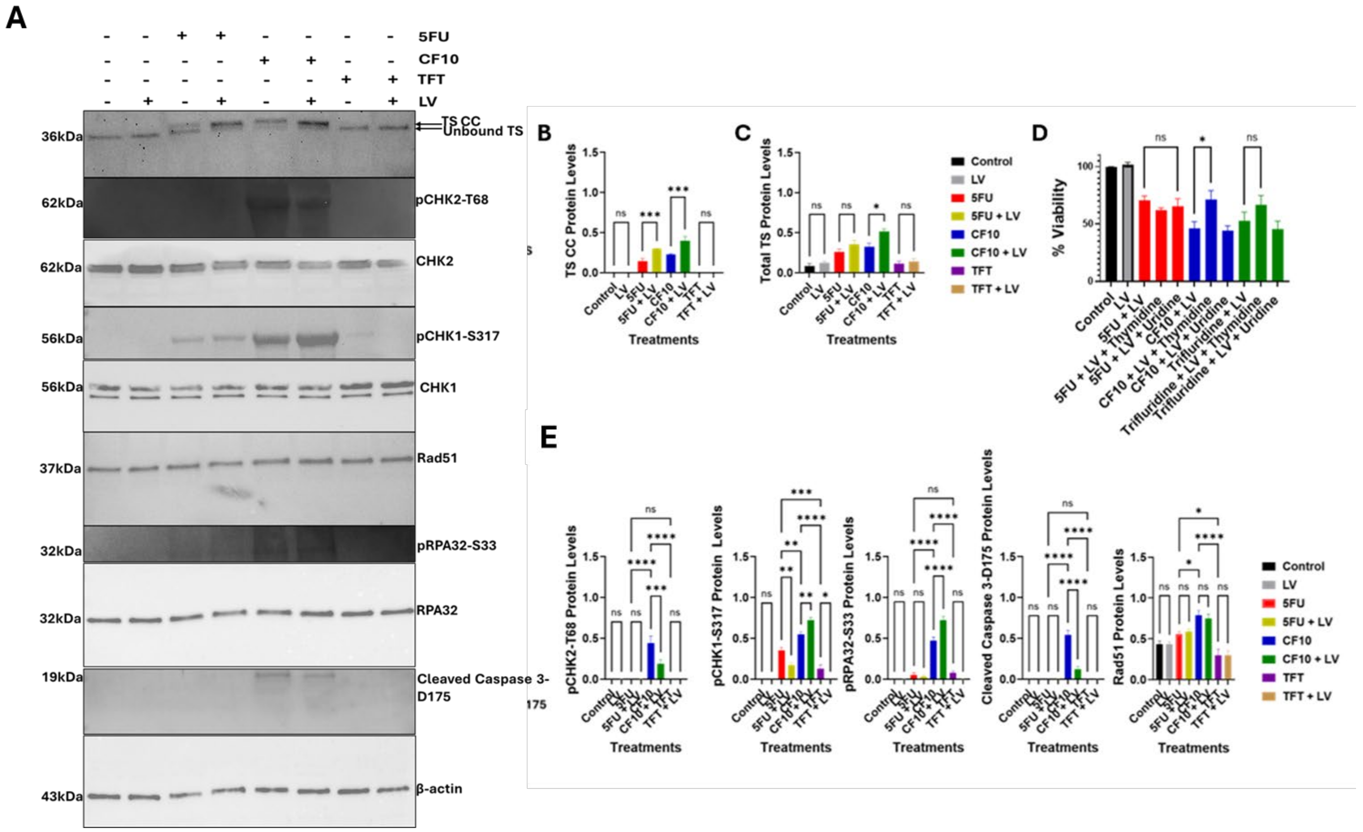

Supplementary Figure S9. CF10 promotes TS classic complex (TS CC) formation and activates the ATR/Chk1 and ATM/Chk2 DNA damage response pathways in HCT15 cells under folate-restricted culture conditions. (**A**) Western blot for 5-FU ± LV, CF10 ± LV, and TFT ± LV detecting the TS CC and unbound TS also detecting protein biomarkers for activation of the ATR/Chk1 (pChk1-S317, pRPA32-S33) and the ATM/Chk2 (pChk2-T68) DNA damage response pathways. Upregulation of the homologous recombination protein Rad 51 involved in DNA double strand break repair and of cleaved caspase 3 is also shown; (**B,C**) Quantification of TS CC and total TS levels; (**D**) effect of thymidine (80 uMol) and uridine (1 mMol) co-treatment on HCT-116 cell viability in FR media for 5-FU+LV (red), CF10+LV (blue), and TFT+LV (green). FP+LV was dosed at IC50 for 48h shown in Figure 2. Experiments done in triplicate ± SEM (***p ≤ 0.0002, **p ≤0.002, *p ≤ 0.03). Decreased cell viability for CF10+LV and TFT+LV is partly reversed by thymidine co-treatment but not for 5-FU+LV. (**E**) quantification of (**A)** for pChk1-S317; pRPA32-S33; Rad 51 levels from densitometry of **(A)**.

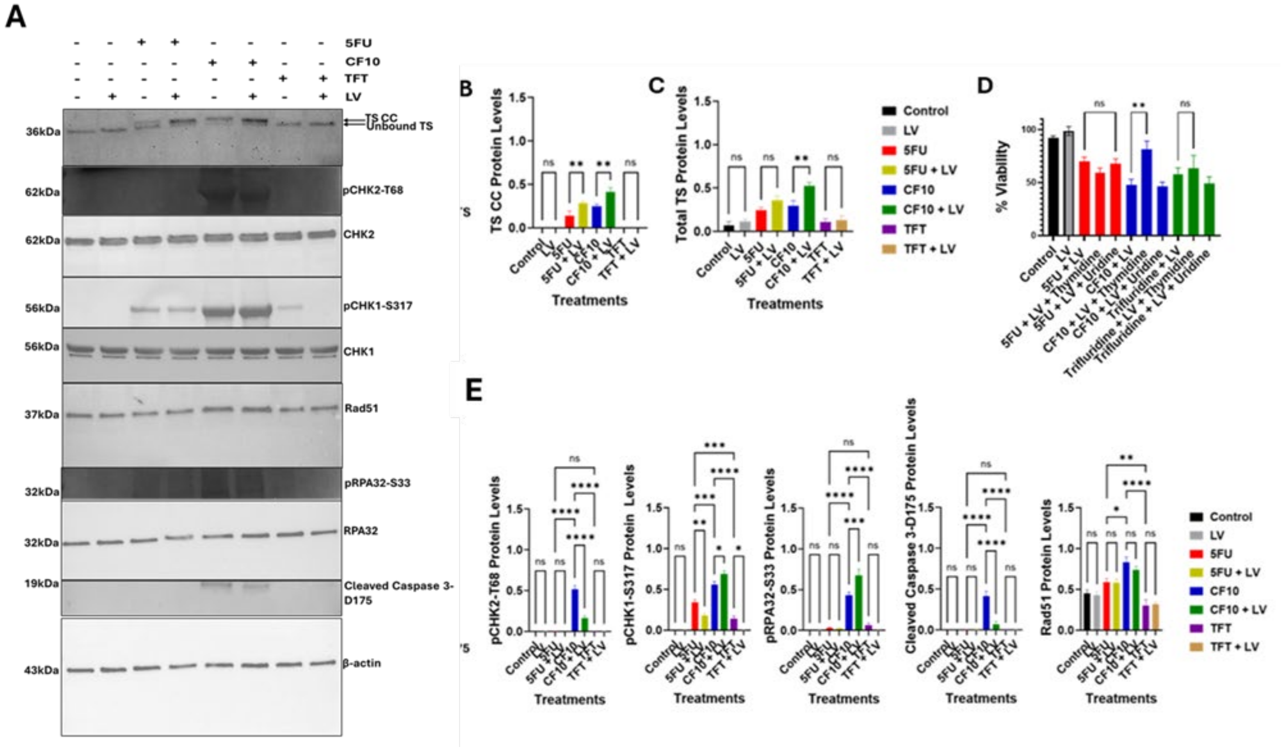

Supplementary Figure S10. CF10 promotes TS classic complex (TS CC) formation and activates the ATR/Chk1 and ATM/Chk2 DNA damage response pathways in MC38 cells under folate-restricted culture conditions. (**A**) Western blot for 5-FU ± LV, CF10 ± LV, and TFT ± LV detecting the TS CC and unbound TS also detecting protein biomarkers for activation of the ATR/Chk1 (pChk1-S317, pRPA32-S33) and the ATM/Chk2 (pChk2-T68) DNA damage response pathways. Upregulation of the homologous recombination protein Rad 51 involved in DNA double strand break repair and of cleaved caspase 3 is also shown; (**B,C**) Quantification of TS CC and total TS levels; (**D**) effect of thymidine (80 uMol) and uridine (1 mMol) co-treatment on HCT-116 cell viability in FR media for 5-FU+LV (red), CF10+LV (blue), and TFT+LV (green). FP+LV was dosed at IC50 for 48h shown in Figure 2. Experiments done in triplicate ± SEM (***p ≤ 0.0002, **p ≤0.002, *p ≤ 0.03). Decreased cell viability for CF10+LV and TFT+LV is partly reversed by thymidine co-treatment but not for 5-FU+LV. (**E**) quantification of **(A)** for pChk1-S317; pRPA32-S33; Rad 51 levels from densitometry of **(A)**.

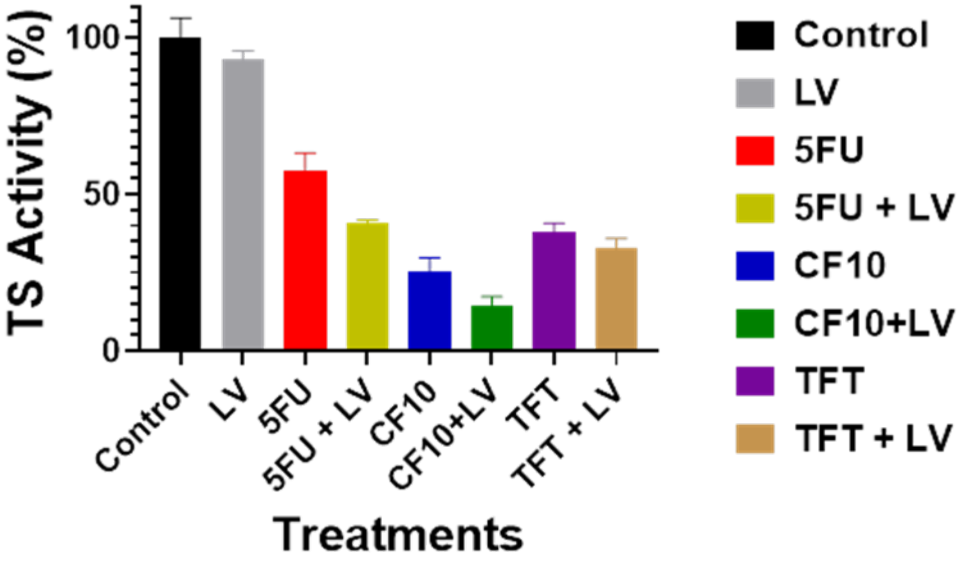

Supplementary Figure S11. CF10 and CF10/LV are effective at reducing TS enzymatic activity. CF10 and LV were used at 10 nM while 5-FU and TFT were used at 100 nM. TS activity was determined using a ^{3}H-release assay by scintillation counting with activity expressed as %-activity relative to untreated controls. Drug treatment was for 24h in HCT-116 cells.

A

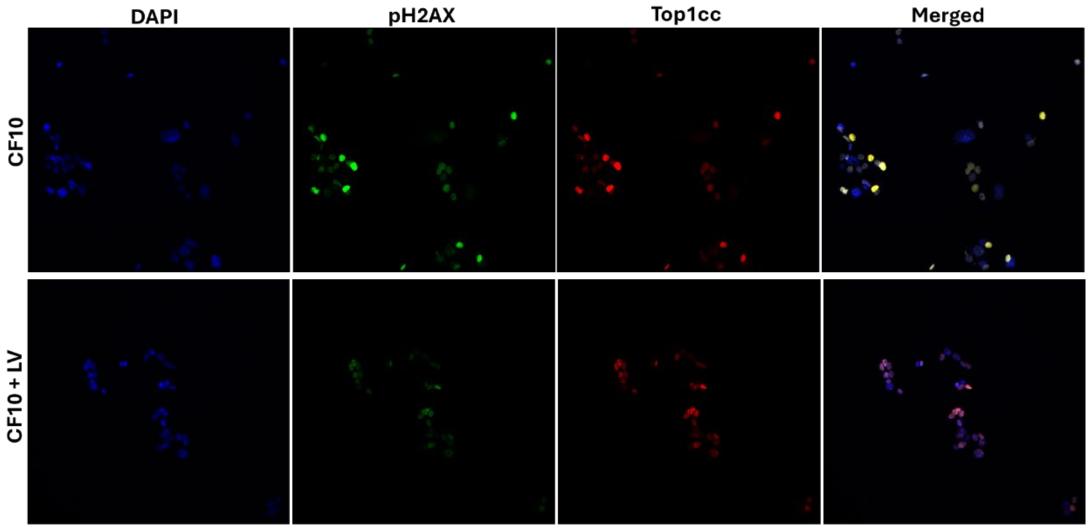

Immunofluorescence imaging of pH2AX and Top1cc in HCT-116 cells following treatment with CF10 or CF10 + LV

B

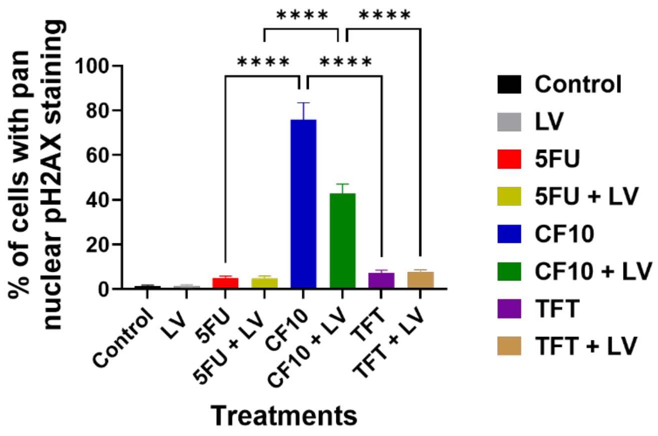

Quantification imaging of pH2AX in HCT-116 cells. ****p ≤ 0.0001

Supplementary Figure S12. CF10 and CF10/LV induce Top1cc in HCT-116 cells (A) and are much more potent than 5-FU±LV or TFT± LV at inducing pH2AX, a marker of DNA DSBs (B).

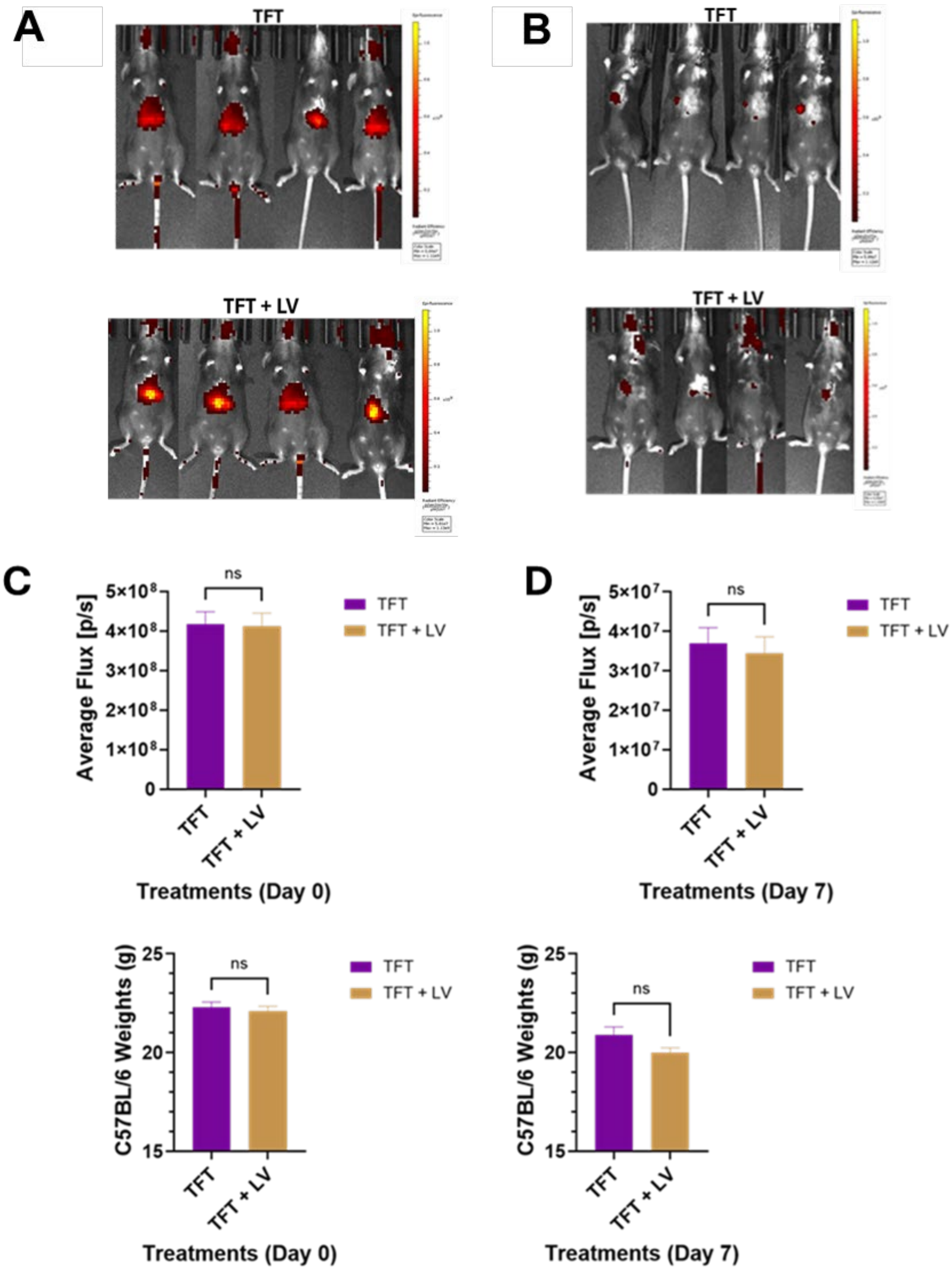

Supplementary Figure S13. TFT and TFT/LV are effective in a liver-metastatic CRC model under conditions of human-like folate levels. **A,B** IVIS images of tumor burden for indicated treatments at day 0 (**A**) and day 7 (**B**) post-treatment. (**C**) Tumor flux pre-treatment (top) and post-treatment (bottom). (**D**) Mice weights pre-treatment (top) and post-treatment (bottom).
