# Supplementary material for: CF10 Displayed Improved Activity Relative to 5-FU in a Mouse CRLM Model Under Conditions of Physiological Folate
Source: Cancers (Basel). 2025 Aug 23;17(17):2739. doi: 10.3390/cancers17172739 (PMC12427396; doi:10.3390/cancers17172739)
Supplement: Supplementary file 1 [file cancers-17-02739-s001.zip › cancers-3743016 - Supplementary Material - WB Original.pdf]

HCT15

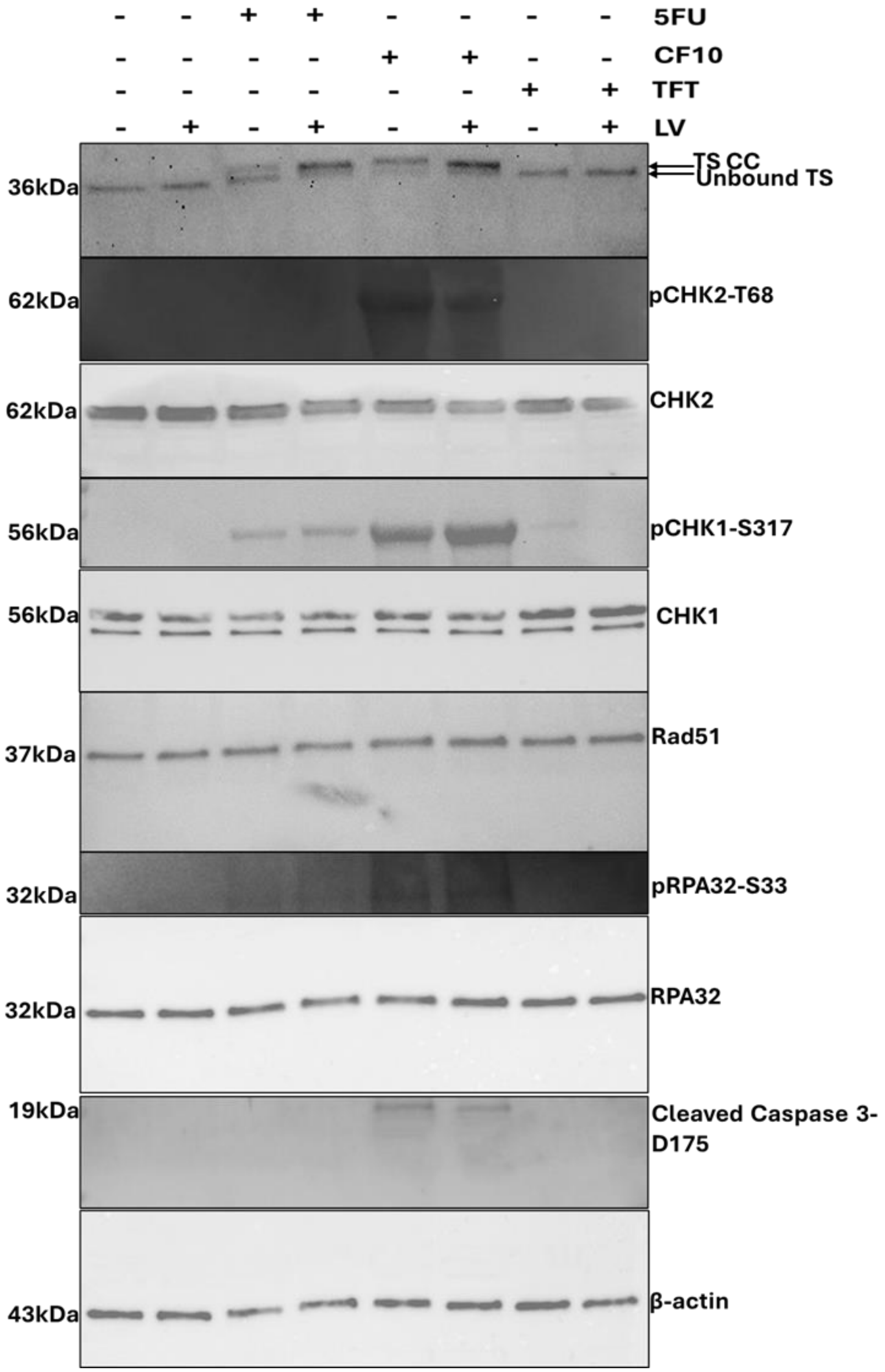

5FU
CF10
TFT
LV
TS CC
Unbound TS
36kDa
pCHK2-T68
62kDa
CHK2
62kDa
pCHK1-S317
56kDa
CHK1
56kDa
Rad51
37kDa
pRPA32-S33
32kDa
RPA32
32kDa
Cleaved Caspase 3-D175
19kDa
β-actin
43kDa

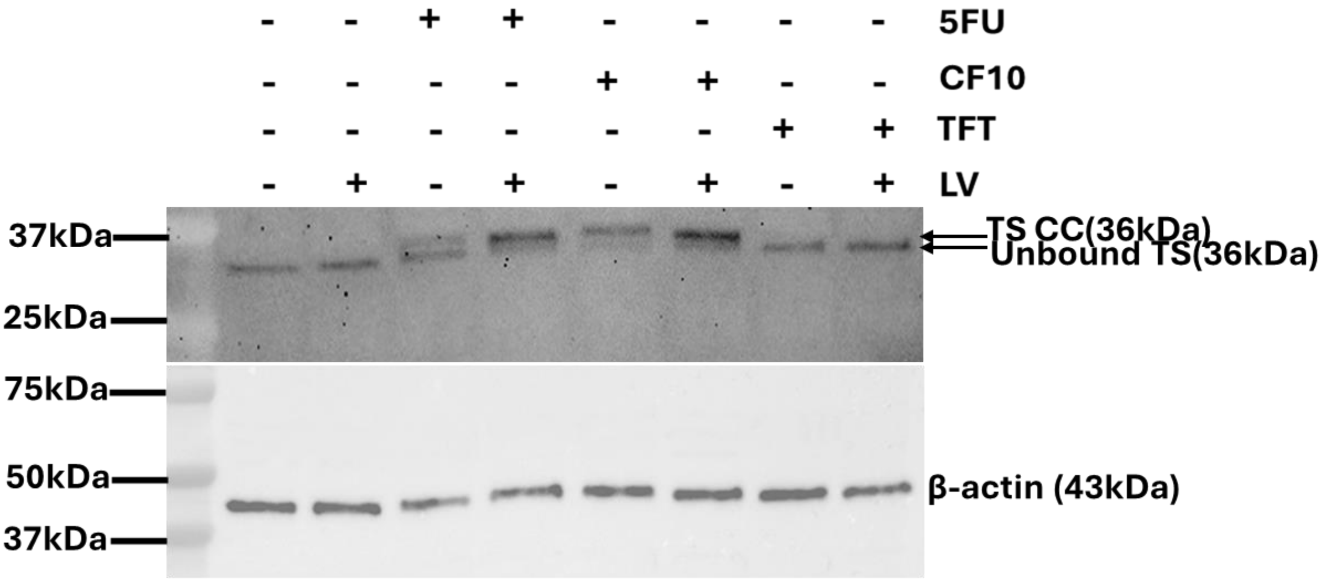

5FU
CF10
TFT
LV
37kDa
25kDa
75kDa
50kDa
37kDa
TS CC(36kDa)
Unbound TS(36kDa)
β-actin (43kDa)

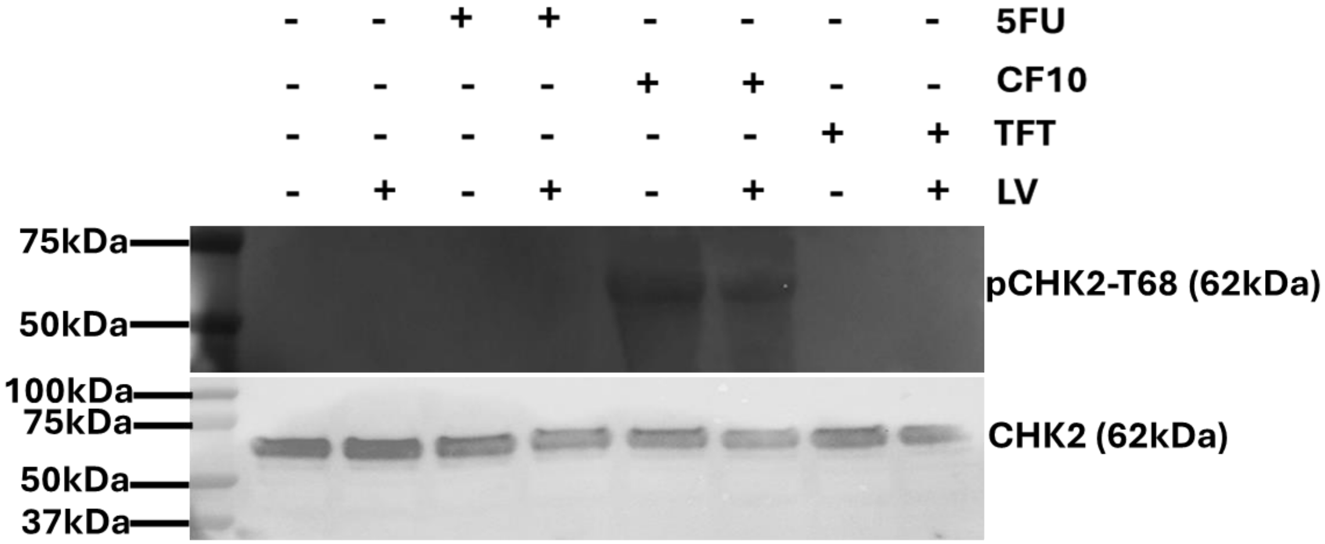

- - + + - - - - 5FU
- - - - + + - - CF10
- - - - - - + + TFT
- + - + - + - + LV
75kDa
50kDa
pCHK2-T68 (62kDa)
100kDa
75kDa
50kDa
37kDa
CHK2 (62kDa)

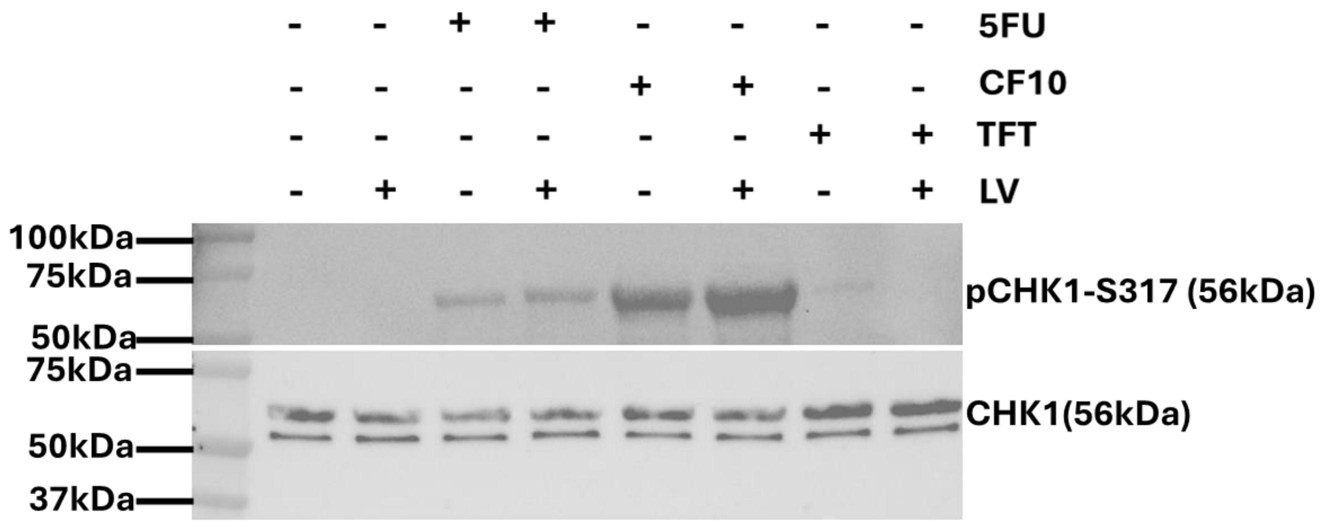

- - + + - - - - 5FU
- - - - + + - - CF10
- - - - - - + + TFT
- + - + - + - + LV
100kDa
75kDa
50kDa
pCHK1-S317 (56kDa)
75kDa
50kDa
37kDa
CHK1(56kDa)

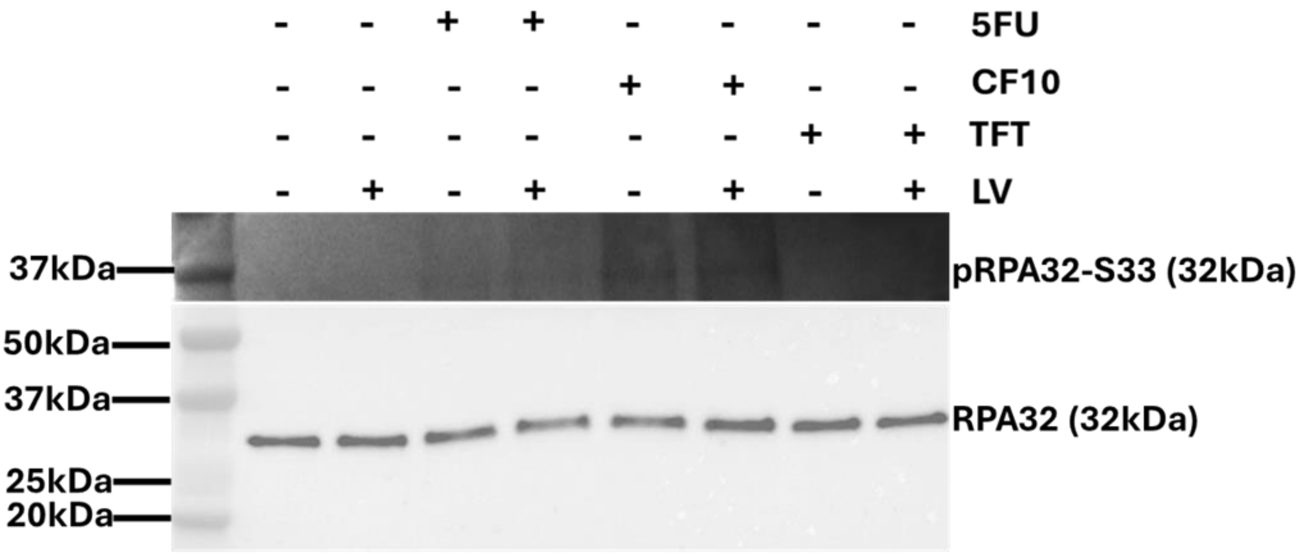
- - + + - - - - 5FU
- - - - + + - - CF10
- - - - - - + + TFT
- + - + - + - + LV
37kDa
pRPA32-S33 (32kDa)
50kDa
37kDa
25kDa
20kDa
RPA32 (32kDa)

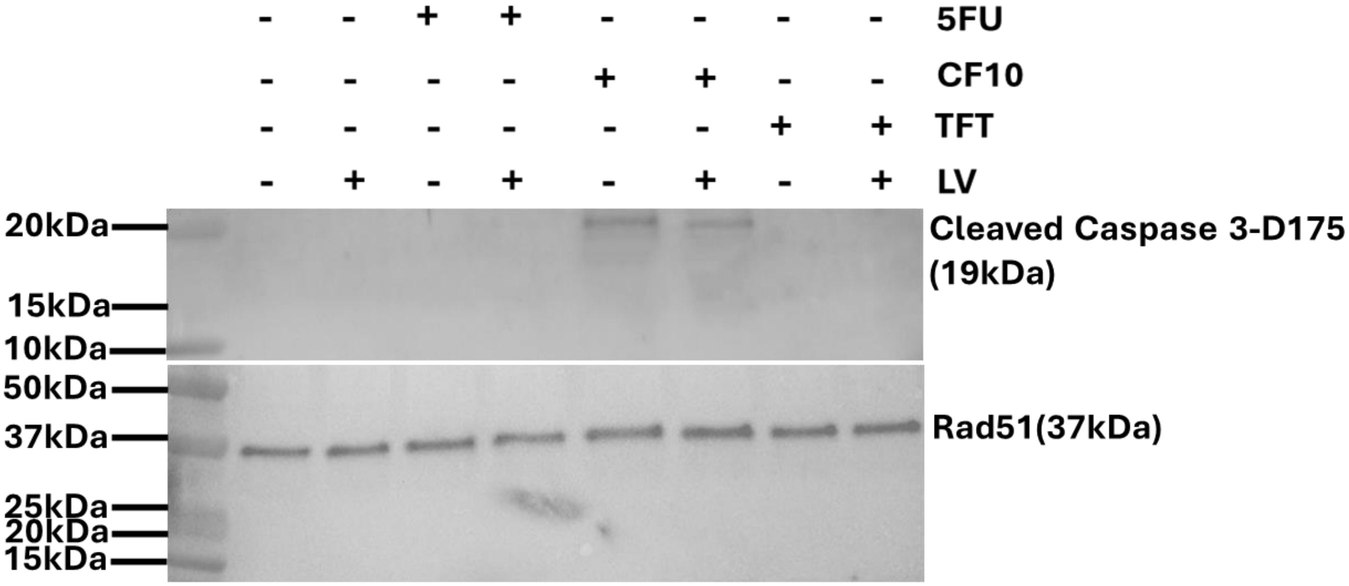
5FU
CF10
TFT
LV
20kDa
15kDa
10kDa
50kDa
37kDa
25kDa
20kDa
15kDa
Cleaved Caspase 3-D175
(19kDa)
Rad51(37kDa)

LS174T

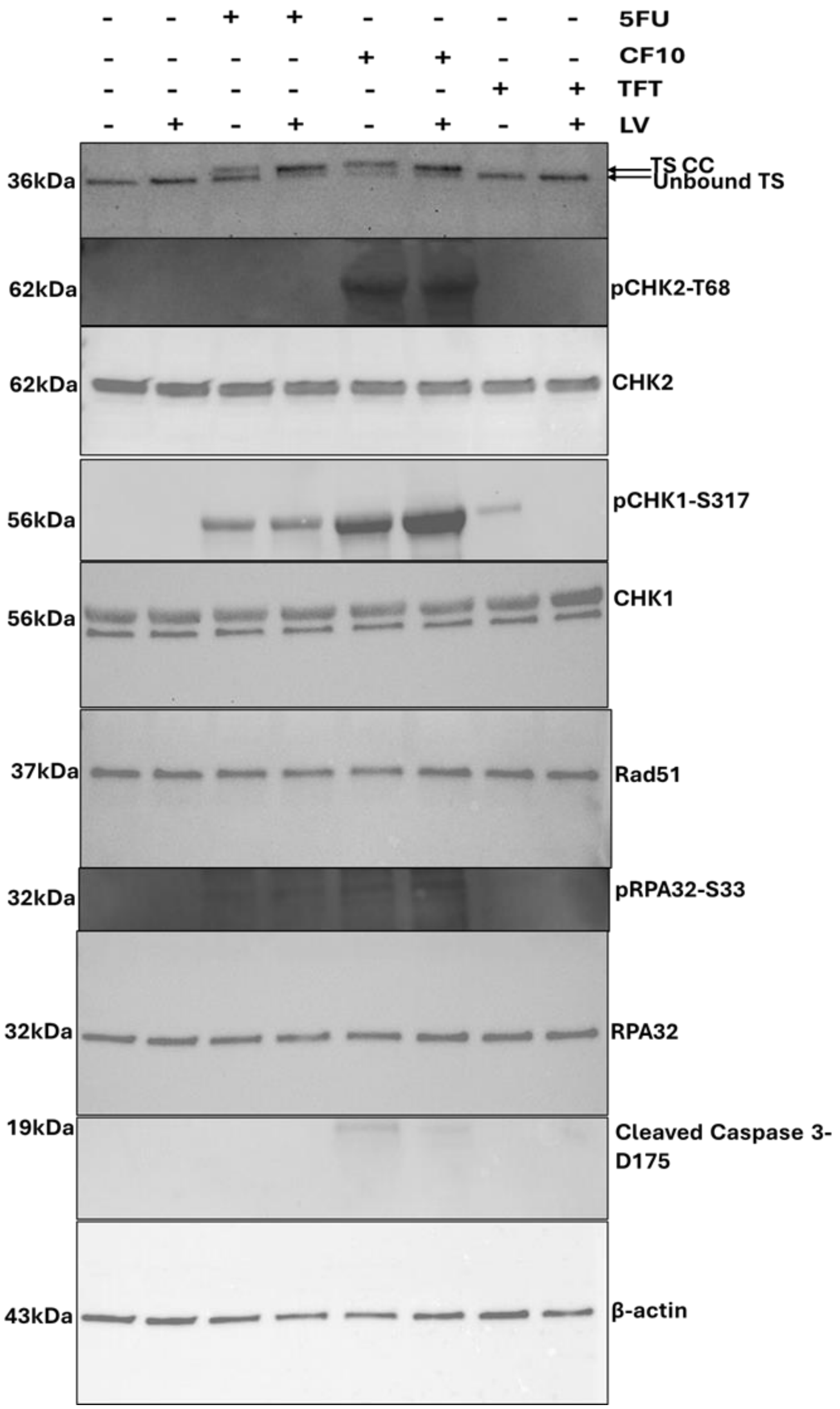

5FU
CF10
TFT
LV
36kDa
TS CC
Unbound TS
62kDa
pCHK2-T68
62kDa
CHK2
56kDa
pCHK1-S317
56kDa
CHK1
37kDa
Rad51
32kDa
pRPA32-S33
32kDa
RPA32
19kDa
Cleaved Caspase 3-D175
43kDa
β-actin

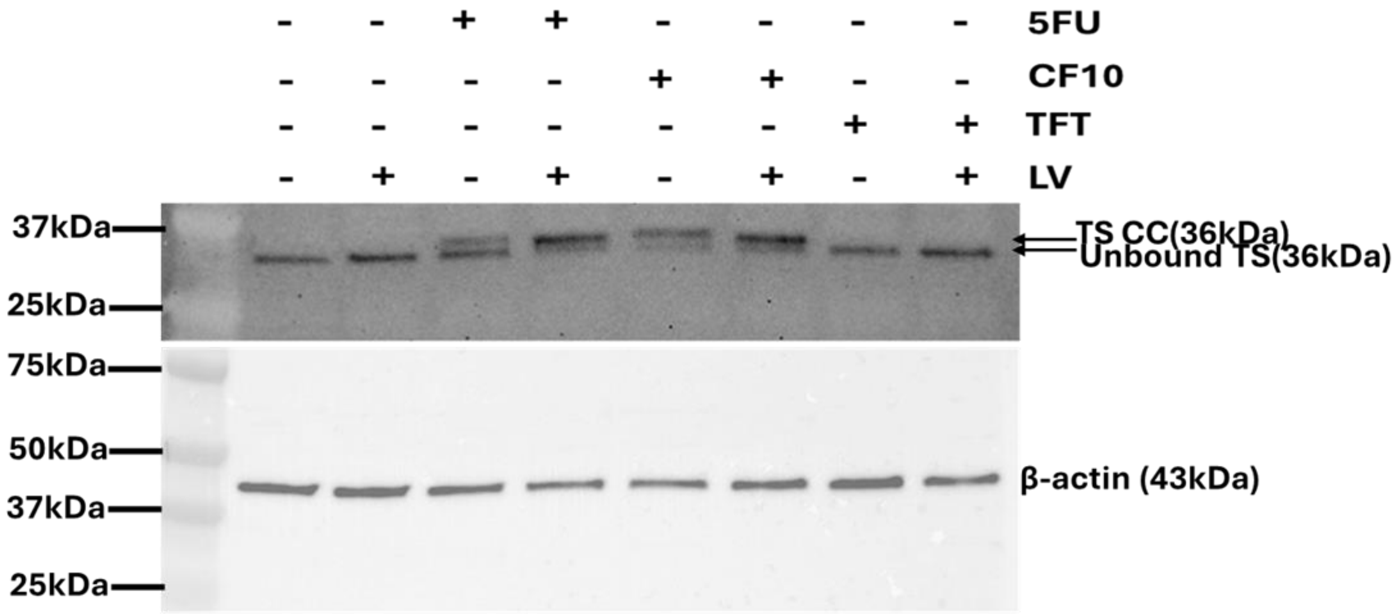

- - + + - - - - 5FU
- - - - + + - - CF10
- - - - - - + + TFT
- + - + - + - + LV
37kDa
25kDa
TS CC(36kDa)
Unbound TS(36kDa)
75kDa
50kDa
37kDa
25kDa
β-actin (43kDa)

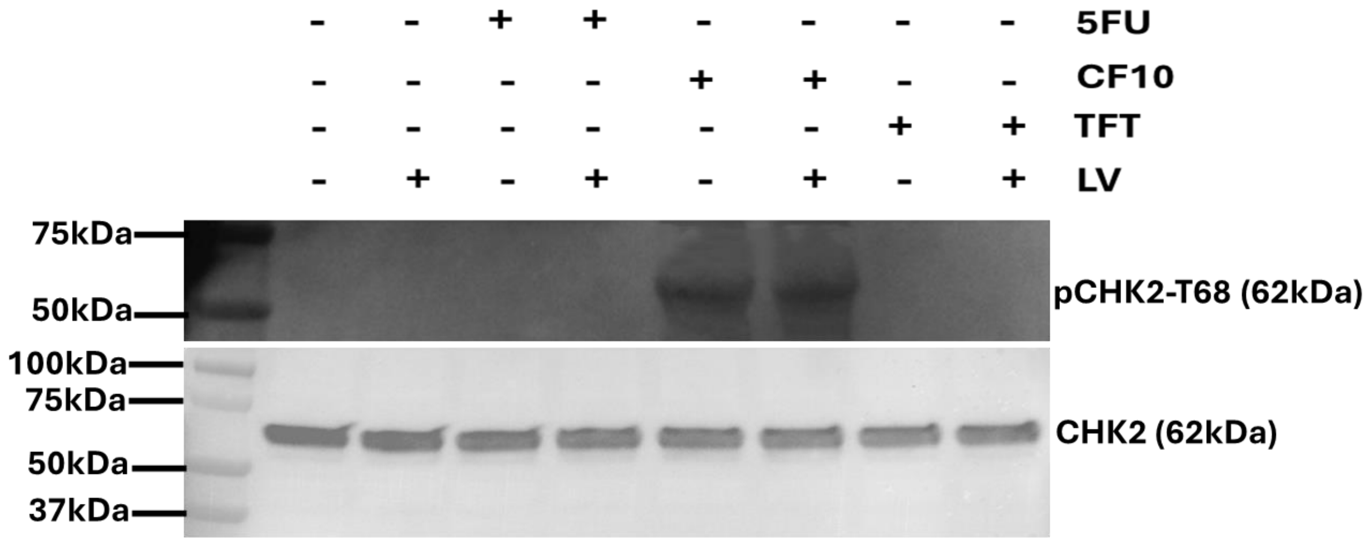

5FU
CF10
TFT
LV
75kDa
50kDa
pCHK2-T68 (62kDa)
100kDa
75kDa
50kDa
37kDa
CHK2 (62kDa)

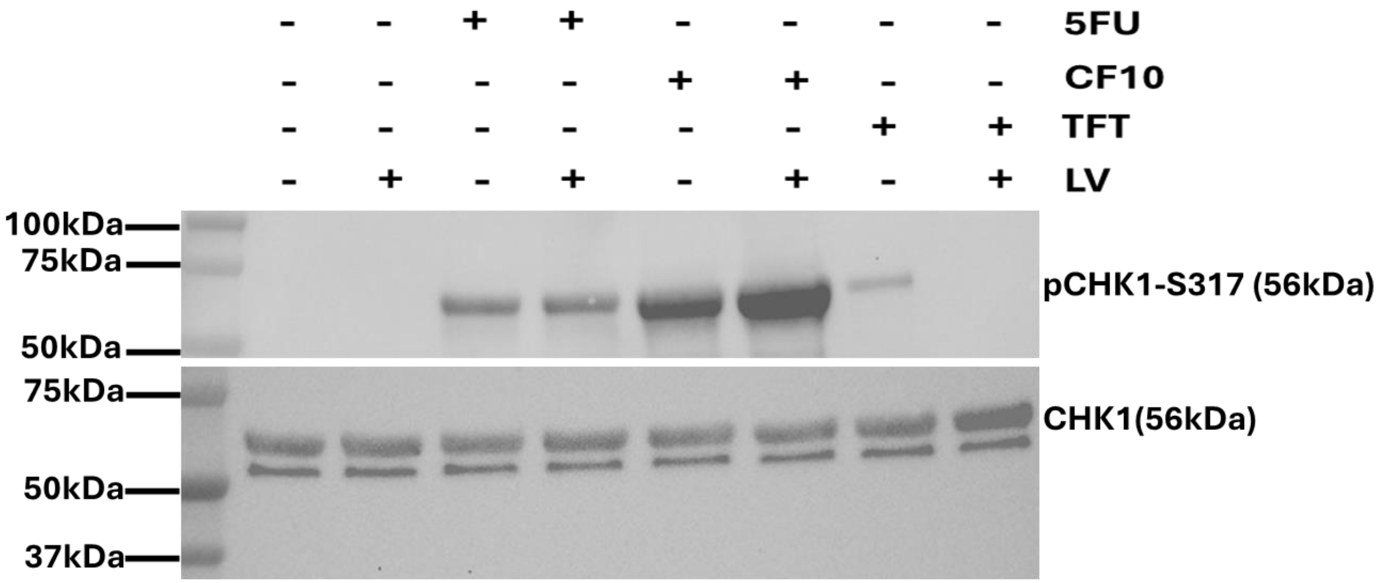
- - + + - - - - 5FU
- - - - + + - - CF10
- - - - - - + + TFT
- + - + - + - + LV
100kDa
75kDa
50kDa
pCHK1-S317 (56kDa)
75kDa
50kDa
37kDa
CHK1(56kDa)

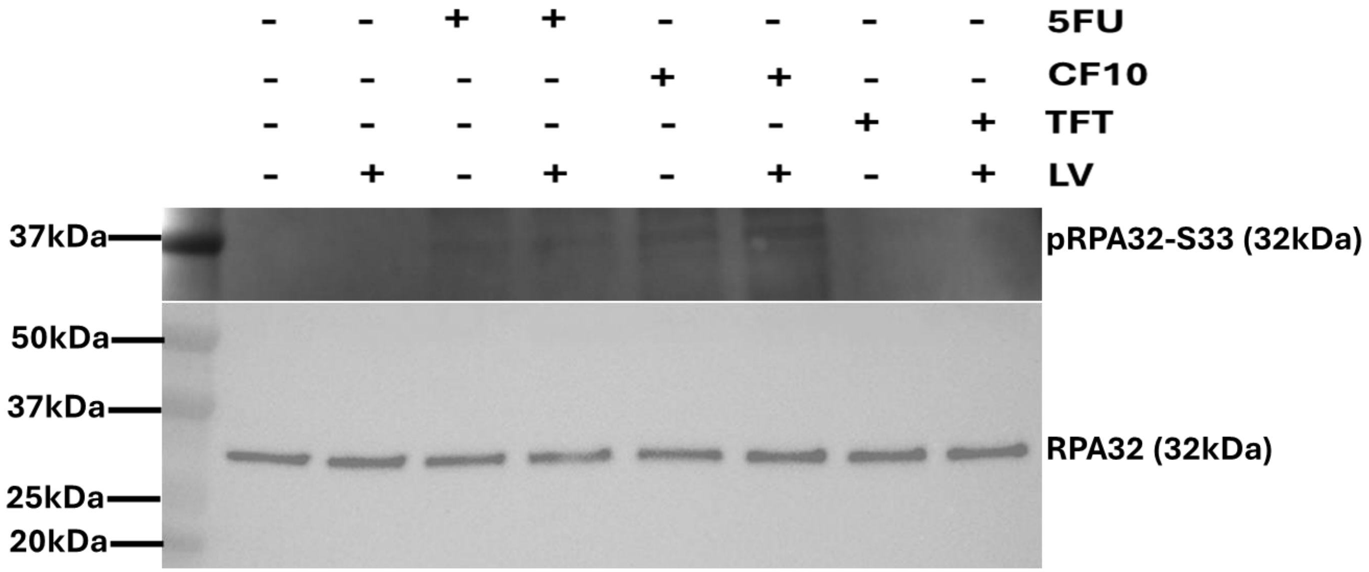
- - + + - - - - 5FU
- - - - + + - - CF10
- - - - - - + + TFT
- + - + - + - + LV
37kDa
pRPA32-S33 (32kDa)
50kDa
37kDa
RPA32 (32kDa)
25kDa
20kDa

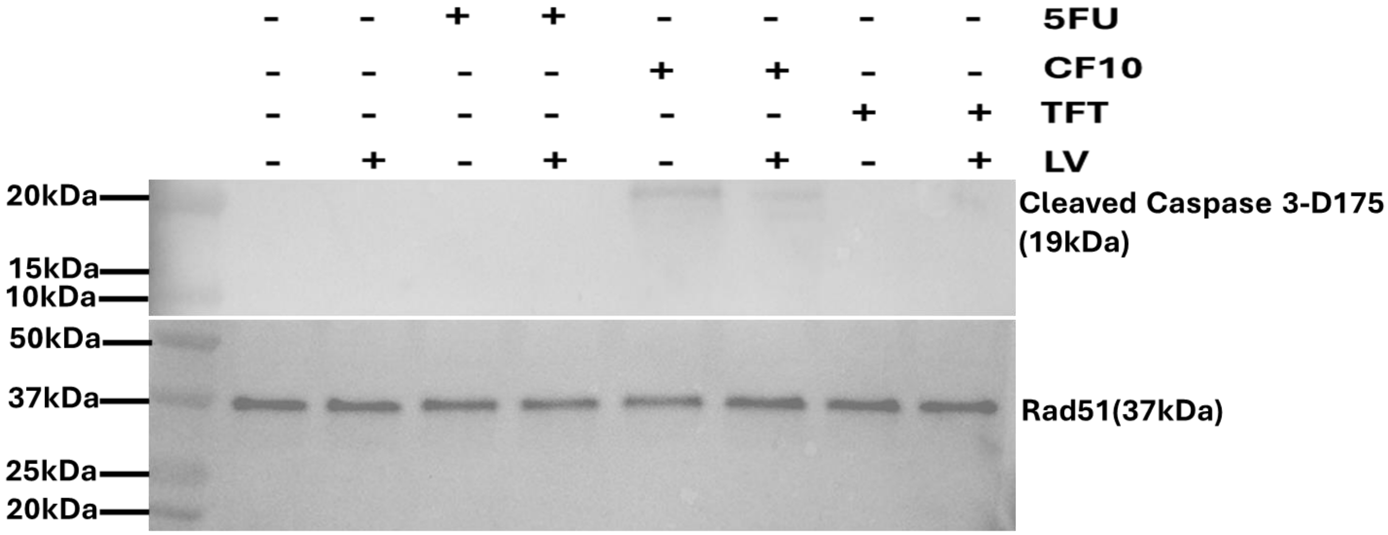
- - + + - - - - 5FU
- - - - + + - - CF10
- - - - - - + + TFT
- + - + - + - + LV
20kDa
15kDa
10kDa
Cleaved Caspase 3-D175 (19kDa)
50kDa
37kDa
25kDa
20kDa
Rad51(37kDa)

MC-38

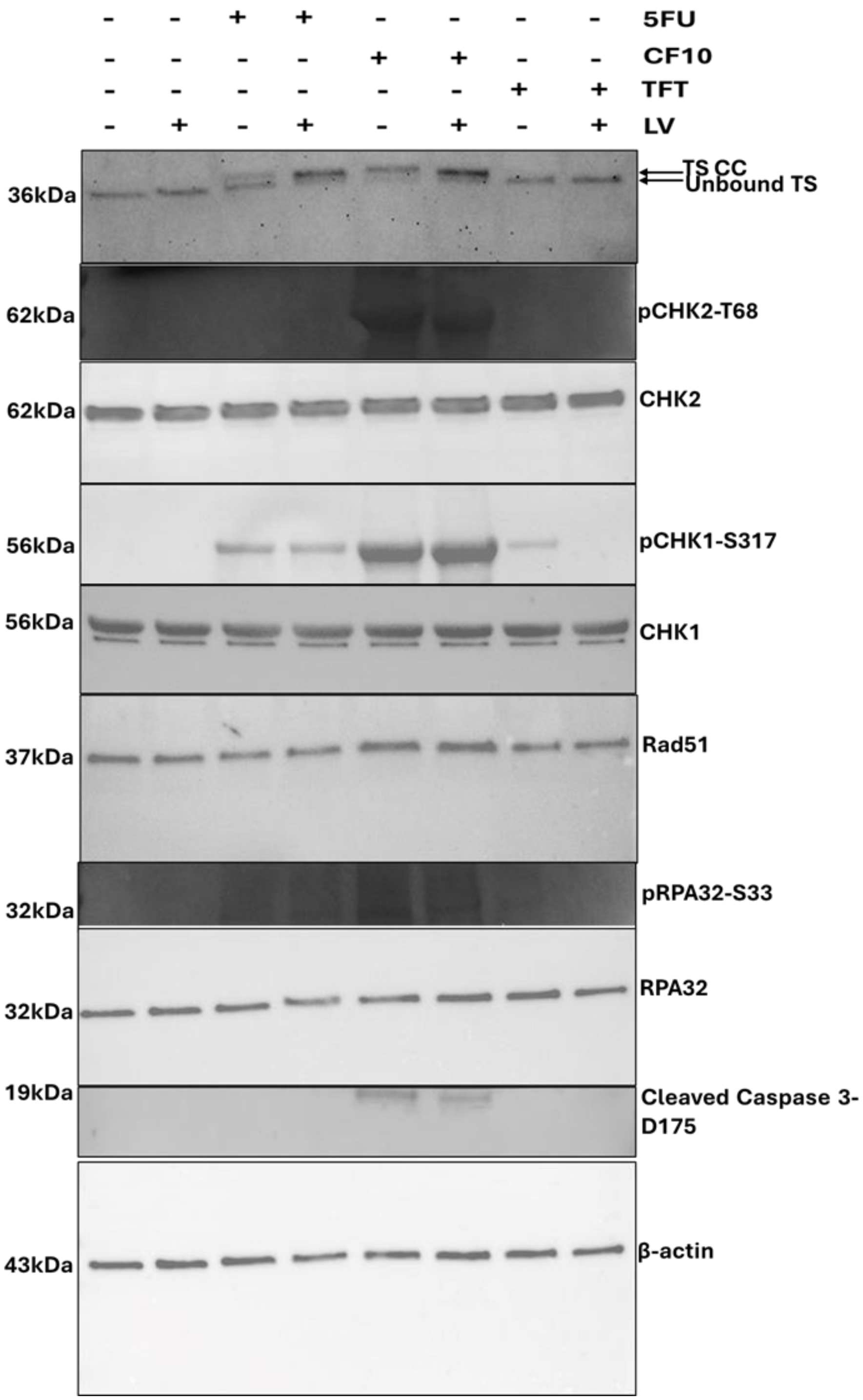

- - + + - - - - 5FU
- - - - + + - - CF10
- - - - - - + + TFT
- + - + - + - + LV
36kDa
TS CC
Unbound TS
62kDa
pCHK2-T68
62kDa
CHK2
56kDa
pCHK1-S317
56kDa
CHK1
37kDa
Rad51
32kDa
pRPA32-S33
32kDa
RPA32
19kDa
Cleaved Caspase 3-D175
43kDa
β-actin

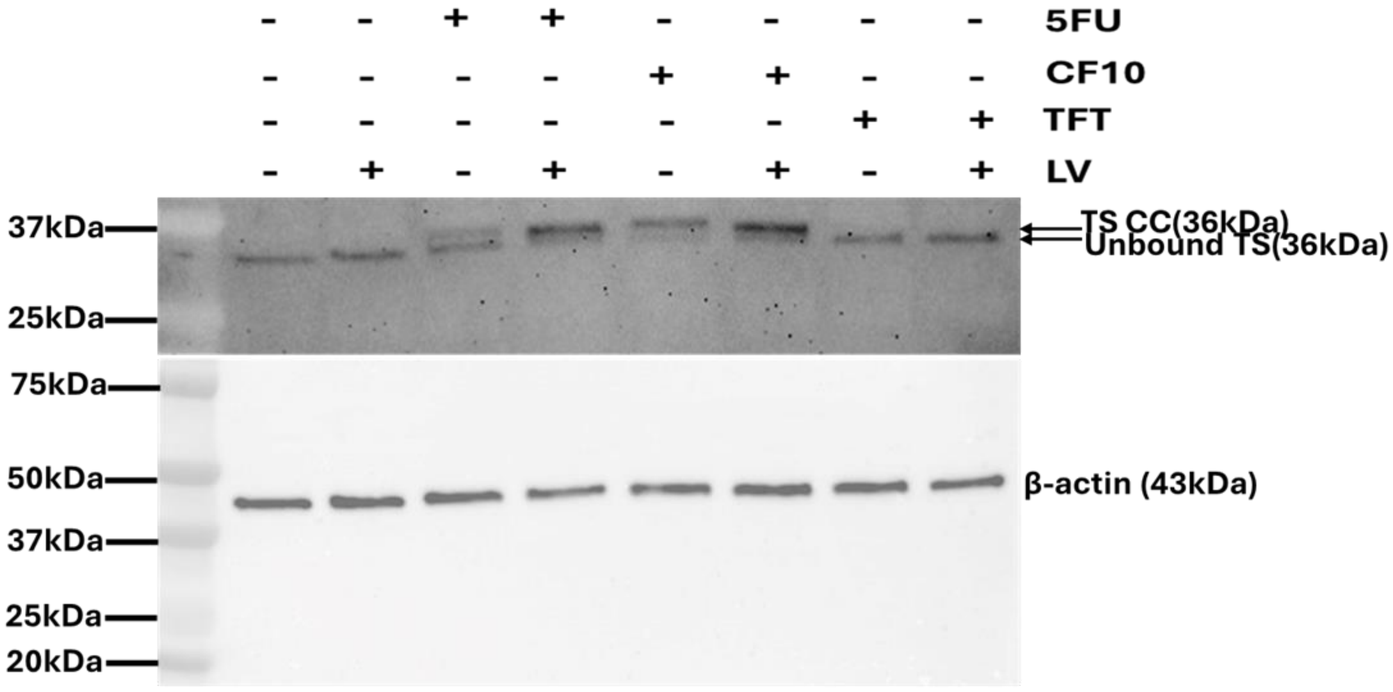

- - + + - - - - 5FU
- - - - + + - - CF10
- - - - - - + + TFT
- + - + - + - + LV
37kDa
25kDa
TS CC(36kDa)
Unbound TS(36kDa)
75kDa
50kDa
37kDa
25kDa
20kDa
β-actin (43kDa)

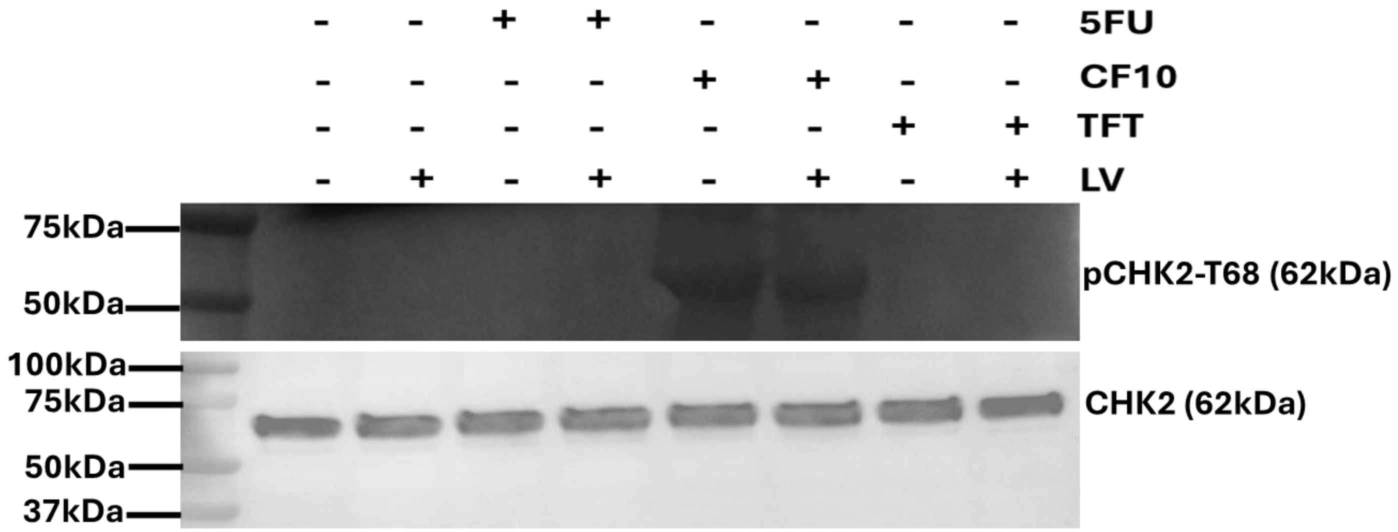

5FU
CF10
TFT
LV
75kDa
50kDa
pCHK2-T68 (62kDa)
100kDa
75kDa
50kDa
37kDa
CHK2 (62kDa)

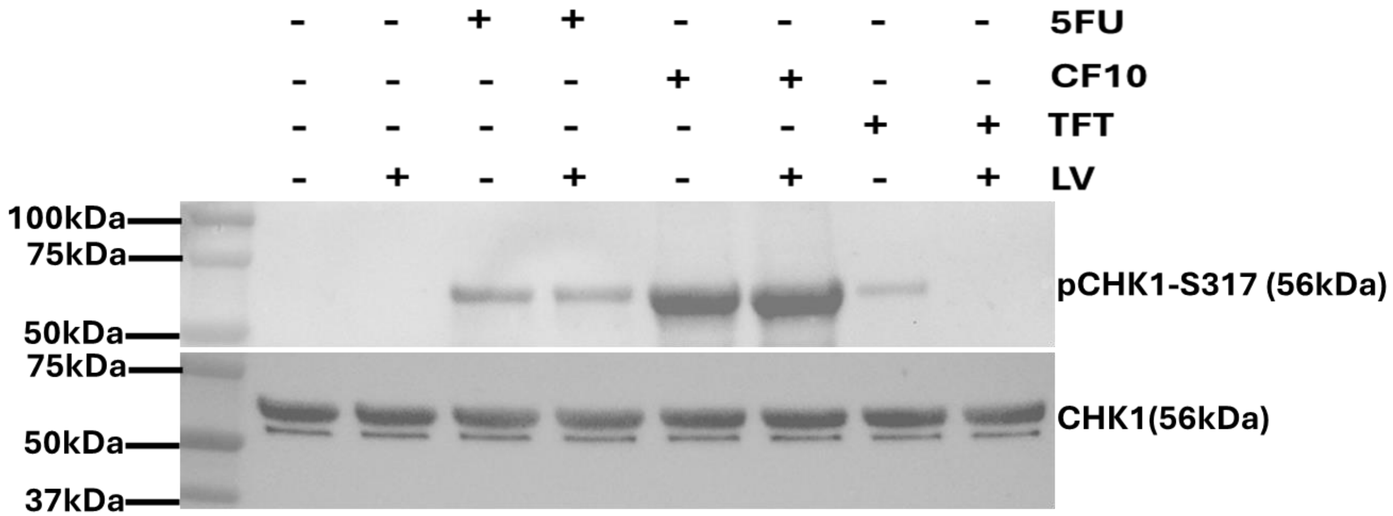

- - + + - - - - 5FU
- - - - + + - - CF10
- - - - - - + + TFT
- + - + - + - + LV
100kDa
75kDa
50kDa
pCHK1-S317 (56kDa)
75kDa
50kDa
37kDa
CHK1(56kDa)

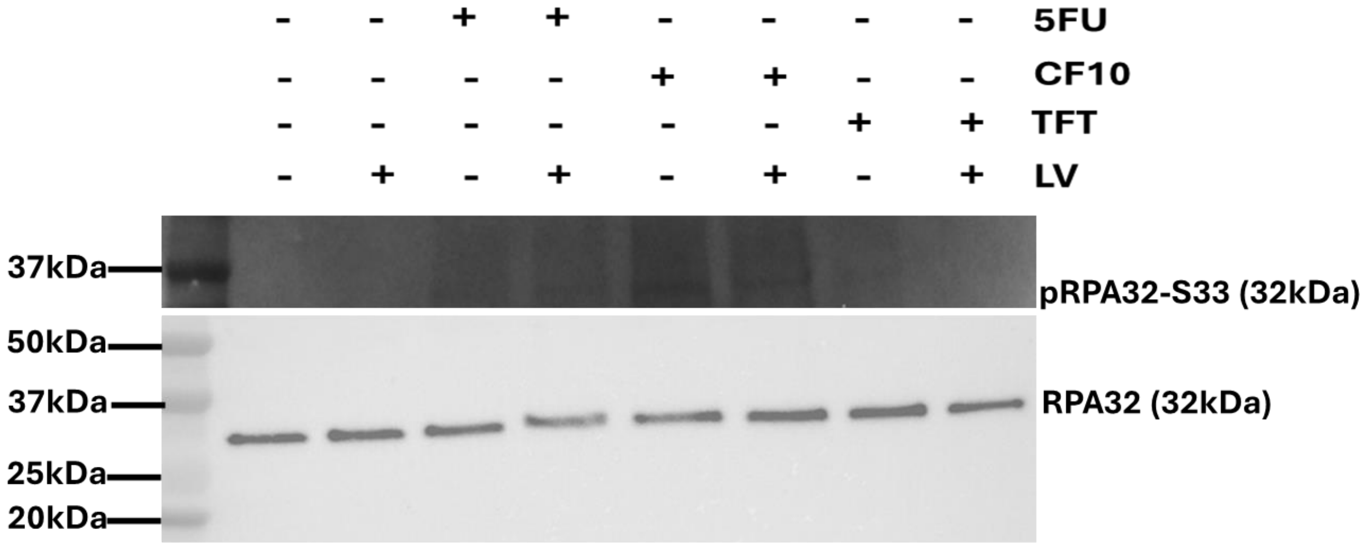

- - + + - - - - 5FU
- - - - + + - - CF10
- - - - - - + + TFT
- + - + - + - + LV
37kDa
pRPA32-S33 (32kDa)
50kDa
37kDa
25kDa
20kDa
RPA32 (32kDa)

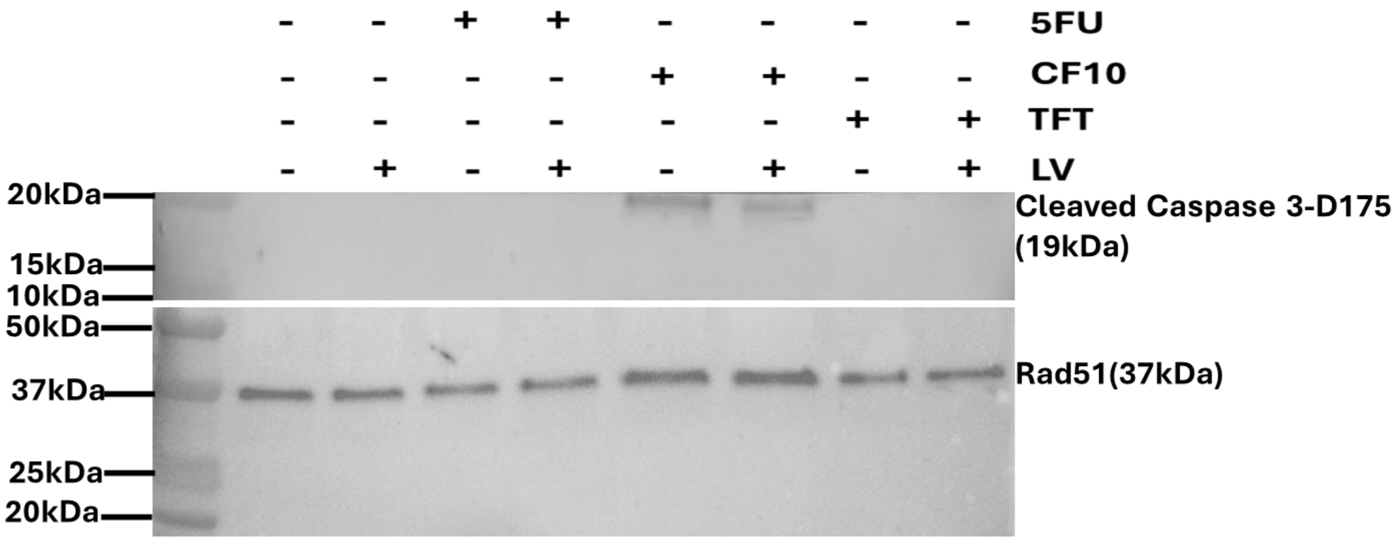

- - + + - - - - 5FU
- - - - + + - - CF10
- - - - - - + + TFT
- + - + - + - + LV
20kDa
15kDa
10kDa
Cleaved Caspase 3-D175
(19kDa)
50kDa
37kDa
25kDa
20kDa
Rad51(37kDa)
