# Supplementary material for: CF10 Displayed Improved Activity Relative to 5-FU in a Mouse CRLM Model Under Conditions of Physiological Folate
Source: Cancers (Basel). 2025 Aug 23;17(17):2739. doi: 10.3390/cancers17172739 (PMC12427396; doi:10.3390/cancers17172739)
Supplement: Supplementary file 1 [file cancers-17-02739-s001.zip › cancers-3743016- Supplementary Tables.pdf]

Supplementary Tables:

CF10 Displays Improved Activity Relative to 5-FU in a Mouse CRLM Model Under Conditions of Physiological Folate

Charles Chidi Okechukwu[1], Xue Ma[2], Wencheng Li[3], Ralph D'Agostino[4], Jr., Matthew G. Rees[5], Melissa M. Ronan[5], Jennifer A. Roth[5], and William H. Gmeiner[1*]

[1]Department of Cancer Biology, Wake Forest University School of Medicine, Winston-Salem, NC 27157 USA;

[2]Department of Orthopedic Surgery. Wake Forest University School of Medicine, Winston-Salem, NC 27157 USA;

[3]Department of Pathology, Wake Forest University School of Medicine, Winston-Salem, NC 27157 USA;

[4]Department of Public Health Sciences and Comprehensive Cancer Center, Wake Forest University School of Medicine, Winston-Salem, NC 27157 USA;

[5]Broad Institute of MIT and Harvard, Cambridge, MA 02142

Running Title: Improved efficacy of CF10 in liver-metastatic CRC

Supplementary Table S1. AUC values for CF10 and 5-FU from the PRISM screen (data are plotted in Fig 1C).

Bowel cell lines PRISM 5FU CF10 AUC 09022024					
**Depmap ID**	**CF10 AUC AUC**	**FLUOROURACIL (BRD:BRD-K24844714-001-24-5) AUC Drug sensitivity AUC (PRISM Repurposing Secondary Screen)**	**Primary Disease**	**Cell Line Name**	**Lineage**
ACH-000982	0.16	0.76	Colorectal Adenocarcinoma	GP2D	Bowel
ACH-000467	0.15	0.83	Colorectal Adenocarcinoma	HCC56	Bowel
ACH-000971	0.34	0.89	Colorectal Adenocarcinoma	HCT116	Bowel
ACH-000997	0.32	0.86	Colorectal Adenocarcinoma	HCT15	Bowel
ACH-000552	0.48	0.87	Colorectal Adenocarcinoma	HT29	Bowel
ACH-000957	0.34	0.83	Colorectal Adenocarcinoma	LS180	Bowel
ACH-000007	0.43	0.65	Colorectal Adenocarcinoma	LS513	Bowel
ACH-000296	0.55	0.87	Colorectal Adenocarcinoma	OUMS23	Bowel
ACH-000565	0.24	0.86	Colorectal Adenocarcinoma	RCM1	Bowel
ACH-000943	0.48	0.86	Colorectal Adenocarcinoma	RKO	Bowel
ACH-000991	0.51	0.80	Colorectal Adenocarcinoma	SNU81	Bowel
ACH-000967	0.58	0.83	Colorectal Adenocarcinoma	SNUC2A	Bowel
ACH-000959	0.22	0.85	Colorectal Adenocarcinoma	SNUC4	Bowel
ACH-000421	0.75	0.89	Colorectal Adenocarcinoma	SW837	Bowel
average	0.40	0.83			

Supplementary Table S2. AUC values for CF10 and Trifluorothymidine from the PRISM screen (data are plotted in Fig. 1D).

CF10 AUC AUC vs TRIFLURIDINE (BRD_BRD-K03243820-001-23-8)…					
**Depmap ID**	**CF10 AUC AUC**	**TRIFLURIDINE (BRD:BRD-K03243820-001-23-8) AUC Drug sensitivity AUC (PRISM Repurposing Secondary Screen)**	**Primary Disease**	**Cell Line Name**	**Lineage**
ACH-000982	0.16	0.47	Colorectal Adenocarcinoma	GP2D	Bowel
ACH-000467	0.15	0.59	Colorectal Adenocarcinoma	HCC56	Bowel
ACH-000971	0.34	0.67	Colorectal Adenocarcinoma	HCT116	Bowel
ACH-000997	0.32	0.63	Colorectal Adenocarcinoma	HCT15	Bowel
ACH-000552	0.48	0.71	Colorectal Adenocarcinoma	HT29	Bowel
ACH-000957	0.34	0.65	Colorectal Adenocarcinoma	LS180	Bowel
ACH-000007	0.43	0.45	Colorectal Adenocarcinoma	LS513	Bowel
ACH-000296	0.55	0.72	Colorectal Adenocarcinoma	OUMS23	Bowel
ACH-000565	0.24	0.55	Colorectal Adenocarcinoma	RCM1	Bowel
ACH-000943	0.48	0.70	Colorectal Adenocarcinoma	RKO	Bowel
ACH-000991	0.51	0.65	Colorectal Adenocarcinoma	SNU81	Bowel
ACH-000967	0.58	0.84	Colorectal Adenocarcinoma	SNUC2A	Bowel
ACH-000959	0.22	0.47	Colorectal Adenocarcinoma	SNUC4	Bowel
ACH-000421	0.75	0.65	Colorectal Adenocarcinoma	SW837	Bowel
average	0.40	0.63			

Supplementary Table S3. AUC values for Trifluorothymidine and 5-FU from the PRISM screen (data are plotted in Supplementary Fig. S2).

	Trifluridine AUC vs 5-FU AUC					
	**Depmap ID**	**FLUOROURACIL (BRD:BRD-K24844714-001-24-5) AUC Drug sensitivity AUC (PRISM Repurposing Secondary Screen)**	**TRIFLURIDINE (BRD:BRD-K03243820-001-23-8) AUC Drug sensitivity AUC (PRISM Repurposing Secondary Screen)**	**Primary Disease**	**Cell Line Name**	**Lineage**
	ACH-000007	0.650901	0.45184	Colorectal Adenocarcinoma	LS513	Bowel
	ACH-000296	0.873327	0.724077	Colorectal Adenocarcinoma	OUMS23	Bowel
	ACH-000421	0.886205	0.653411	Colorectal Adenocarcinoma	SW837	Bowel
	ACH-000467	0.834067	0.592045	Colorectal Adenocarcinoma	HCC56	Bowel
	ACH-000552	0.873021	0.713029	Colorectal Adenocarcinoma	HT29	Bowel
	ACH-000565	0.860207	0.552101	Colorectal Adenocarcinoma	RCM1	Bowel
	ACH-000943	0.864467	0.700899	Colorectal Adenocarcinoma	RKO	Bowel
	ACH-000957	0.834016	0.648421	Colorectal Adenocarcinoma	LS180	Bowel
	ACH-000958	0.871653	0.680961	Colorectal Adenocarcinoma	SW48	Bowel
	ACH-000959	0.846091	0.467151	Colorectal Adenocarcinoma	SNUC4	Bowel
	ACH-000967	0.825733	0.837522	Colorectal Adenocarcinoma	SNUC2A	Bowel
	ACH-000971	0.887555	0.674503	Colorectal Adenocarcinoma	HCT116	Bowel
	ACH-000982	0.757573	0.472937	Colorectal Adenocarcinoma	GP2D	Bowel
	ACH-000991	0.7977	0.652069	Colorectal Adenocarcinoma	SNU81	Bowel
	ACH-000997	0.857639	0.633404	Colorectal Adenocarcinoma	HCT15	Bowel

Supplementary Table S4. Summary of p-values for the correlations of SN-38 with indicated drugs from the Broad Institute PRISM screen (all cell lines). Data plots are shown in Supplementary Fig S3.

Drug	p- value vs SN-38
CPT	5e-37
CF10	5e-24
TFT	1.1e-16
5-FU	1.1e-4

Supplementary Table S5. Summary of GI50 values for LS174T CRC cell line for CF10, 5FU, TFT, and ± LV combination. Plots of data are shown in Supplementary Fig. S5.

FR Drug	Media	IC_{50} (µM) ± SEM	IC_{50} (µM) + LV (1µM) ± SEM	IC_{50} (µM) + LV (10µM) ± SEM	LV Effect (1µM)	LV Effect (10µM)
5FU	DMEM	4.54 ± 0.0125	3.72 ± 0.000999	1.71 ± 0.0101	1.22	2.66
5FU	FR	1.84 ± 0.0165	1.48 ± 0.0126	0.770 ± 0.0137	1.25	2.39
CF10	DMEM	0.0145 ± 0.000124	0.0139 ± 0.000122	0.00697 ± 0.000120	1.04	2.08
CF10	FR	0.0120 ± 0.000127	0.00985 ± 0.000136	0.00602 ± 0.00138	1.21	1.99
TFT	DMEM	2.16 ± 0.0120	2.01 ± 0.0134	1.33 ± 0.0129	1.08	1.62
TFT	FR	1.73 ± 0.0138	1.35 ± 0.0136	0.733 ± 0.0143	1.28	2.36

Supplementary Table S6. Summary of GI50 values for HCT15 CRC cell line for CF10, 5FU, TFT, and ± LV combination. Plots of data are shown in Supplementary Fig. S6.

FR Drug	Media	IC_{50} (µM) ± SEM	IC_{50} (µM) + LV (1µM) ± SEM	IC_{50} (µM) + LV (10µM) ± SEM	LV Effect (1µM)	LV Effect (10µM)
5FU	DMEM	5.07 ± 0.0124	4.36 ± 0.0110	2.51 ± 0.00992	1.16	2.02
5FU	FR	2.20 ± 0.0146	1.97 ± 0.0120	1.12 ± 0.0125	1.12	1.95
CF10	DMEM	0.0521 ± 0.0000990	0.0411 ± 0.0000922	0.0284 ± 0.0000887	1.27	1.84
CF10	FR	0.0455 ± 0.0000910	0.0364 ± 0.000102	0.0220 ± 0.0000978	1.25	2.07
TFT	DMEM	11.12 ± 0.0104	7.85 ± 0.0109	5.34 ± 0.0105	1.42	2.08
TFT	FR	8.62 ± 0.0119	8.35 ± 0.0122	6.31 ± 0.0126	1.03	1.37

Supplementary Table S7. Summary of GI50 values for MC38 CRC cell line for CF10, 5FU, TFT, and ± LV combination. Plots of data are shown in Supplementary Fig. S7.

FR Drug	Media	IC_{50} (µM) ± SEM	IC_{50} (µM) + LV (1µM) ± SEM	IC_{50} (µM) + LV (10µM) ± SEM	LV Effect (1µM)	LV Effect (10µM)
5FU	DMEM	4.98 ± 0.0124	3.66 ± 0.00989	1.50 ± 0.0104	1.36	3.32
5FU	FR	1.49 ± 0.0179	0.946 ± 0.0138	0.480 ± 0.0160	1.58	3.10
CF10	DMEM	0.0117 ± 0.000127	0.0100 ± 0.000132	0.00562 ± 0.000140	1.17	2.08
CF10	FR	0.0108 ± 0.000130	0.00896 ± 0.000139	0.00497 ± 0.000145	1.21	2.17
TFT	DMEM	2.56 ± 0.0117	2.13 ± 0.0132	1.18 ± 0.0132	1.20	2.17
TFT	FR	1.35 ± 0.0115	0.950 ± 0.0145	0.488 ± 0.0151	1.42	2.77

Supplementary Table S8. Least squares means analysis of change in IVIS flux intensities during treatment (Delta IVIS Flux – 7 groups.

group	Mean delta_trt
**Control**	385500000
**CF10**	-402775000
**CF10LV**	-416250000
**FU**	-204500000
**FULV**	-298500000
**LV**	216000000
**TFT**	-404100000
**TFT+LV**	-398025000

**Pairwise Comparison – Using Bonferroni Adjusted P-value for significance (P<0.0017 is statistically significant (yellow)) Dependent Variable: delta_trt T-statistics and P-values Shown**								
**i/j**	**Control**	**CF10**	**CF10LV**	**FU**	**FULV**	**LV**	**TFT**	**TFT+LV**
**Control**		18.1<br><.0001	18.45<br><.0001	13.58<br><.0001	15.7<br><.0001	3.9<br>0.0007	18.17<br><.0001	18.03<br><.0001
**CF10**	-18.1<br><.0001		0.31<br>0.7591	-4.56<br>0.0001	-2.40<br>0.0245	-14.24<br><.0001	0.03<br>0.97	-0.109<br>0.91
**CF10LV**	-18.45<br><.0001	-0.31<br>0.75		-4.87<br><.0001	-2.71<br>0.0122	-14.55<br><.0001	-0.279<br>0.78	-0.419<br>0.67
**FU**	-13.58<br><.0001	4.564<br>0.0001	4.87<br><.0001		2.16<br>0.0406	-9.68<br><.0001	4.59<br>0.0001	4.45<br>0.0002
**FULV**	-15.7<br><.0001	2.40<br>0.0245	2.71<br>0.0122	-2.16<br>0.0406		-11.84<br><.0001	2.43<br>0.0229	2.29<br>0.0310
**LV**	-3.9<br>0.0007	14.24<br><.0001	14.55<br><.0001	9.68<br><.0001	11.84<br><.0001		14.27<br><.0001	14.13<br><.0001
**TFT**	-18.17<br><.0001	-0.03<br>0.97	0.279<br>0.78	-4.59<br>0.0001	-2.43<br>0.0229	-14.27<br><.0001		-0.139<br>0.889
**TFT+LV**	-18.03<br><.0001	0.109<br>0.91	0.419<br>0.67	-4.45<br>0.0002	-2.29<br>0.0310	-14.13<br><.0001	0.139<br>0.889	

Supplementary Table S9. Least squares means analysis of change in weight during treatment. Delta weight – 7 groups.

group	Mean delta_weight
Control	1.33750000
CF10	0.72500000
CF10LV	-0.14000000
FU	-5.41250000
FULV	-3.79250000
LV	-0.78500000
TFT	-1.62500000
TFT+LV	-2.12500000

Pairwise Comparison – Using Bonferroni Adjusted P-value for significance (P<0.0017 is statistically significant (yellow)) Dependent Variable: delta_weight T-statistics and P-values Shown								
i/j	Control	CF10	CF10LV	FU	FULV	LV	TFT	TFT+LV
Control		1.53<br>0.1377	3.704662<br>0.0011	16.92485<br><.0001	12.86289<br><.0001	5.321925<br><.0001	7.428129<br><.0001	8.681822<br><.0001
CF10	-1.53<br>0.1377		2.168888<br>0.0402	15.38908<br><.0001	11.32711<br><.0001	3.786152<br>0.0009	5.892355<br><.0001	7.146048<br><.0001
CF10LV	-3.70466<br>0.0011	-2.16889<br>0.0402		13.22019<br><.0001	9.158225<br><.0001	1.617264<br>0.1189	3.723467<br>0.0011	4.97716<br><.0001
FU	-16.9249<br><.0001	-15.3891<br><.0001	-13.2202<br><.0001		-4.06196<br>0.0005	-11.6029<br><.0001	-9.49672<br><.0001	-8.24303<br><.0001
FULV	-12.8629<br><.0001	-11.3271<br><.0001	-9.15822<br><.0001	4.061964<br>0.0005		-7.54096<br><.0001	-5.43476<br><.0001	-4.18106<br>0.0003
LV	-5.32193<br><.0001	-3.78615<br>0.0009	-1.61726<br>0.1189	11.60293<br><.0001	7.540961<br><.0001		2.106204<br>0.0458	3.359896<br>0.0026
TFT	-7.42813<br><.0001	-5.89236<br><.0001	-3.72347<br>0.0011	9.496722<br><.0001	5.434758<br><.0001	-2.1062<br>0.0458		1.253693<br>0.2220
TFT+LV	-8.68182<br><.0001	-7.14605<br><.0001	-4.97716<br><.0001	8.243029<br><.0001	4.181065<br>0.0003	-3.3599<br>0.0026	-1.25369<br>0.2220	

Supplementary Table S10. Summary of pathological review of liver scarring/inflammation and % tumor burden

**Treatment Group**	**Early Scarring/ Inflammation**	**% Tumor Burden**
Control	Negative	97.00 ± 3.22
LV	Positive	41.00 ± 9.07
5FU	Positive	21.33 ± 3.48
5FU + LV	Positive	11.33 ± 2.40
CF10	Negative	0.00 ± 0.00
CF10 + LV	Negative	0.00 ± 0.00
TFT	Positive	7.00 ± 1.73
TFT + LV	Positive	4.93 ± 1.55
