# Supplementary figures and images for: CF10 Displayed Improved Activity Relative to 5-FU in a Mouse CRLM Model Under Conditions of Physiological Folate
Source: Cancers (Basel). 2025 Aug 23;17(17):2739. doi: 10.3390/cancers17172739 (PMC12427396; doi:10.3390/cancers17172739)

|   |   |   |   |   |   |   |   |      |
|---|---|---|---|---|---|---|---|------|
| - | - | + | + | - | - | - | - | 5FU  |
| - | - | - | - | + | + | - | - | CF10 |
| - | - | - | - | - | - | + | + | TFT  |
| - | + | - | + | - | + | - | + | LV   |

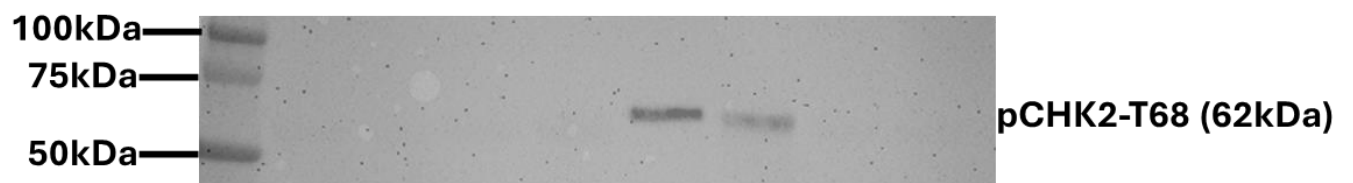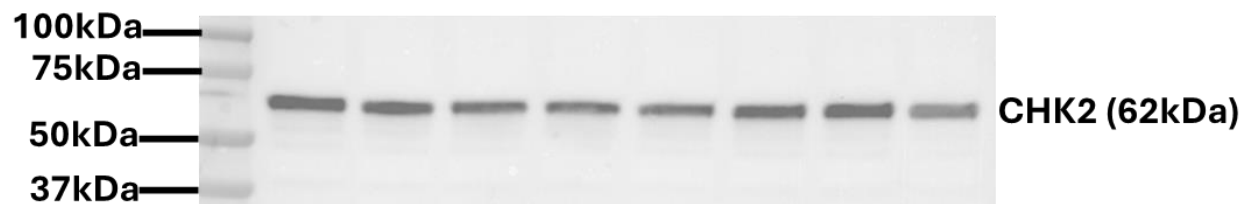

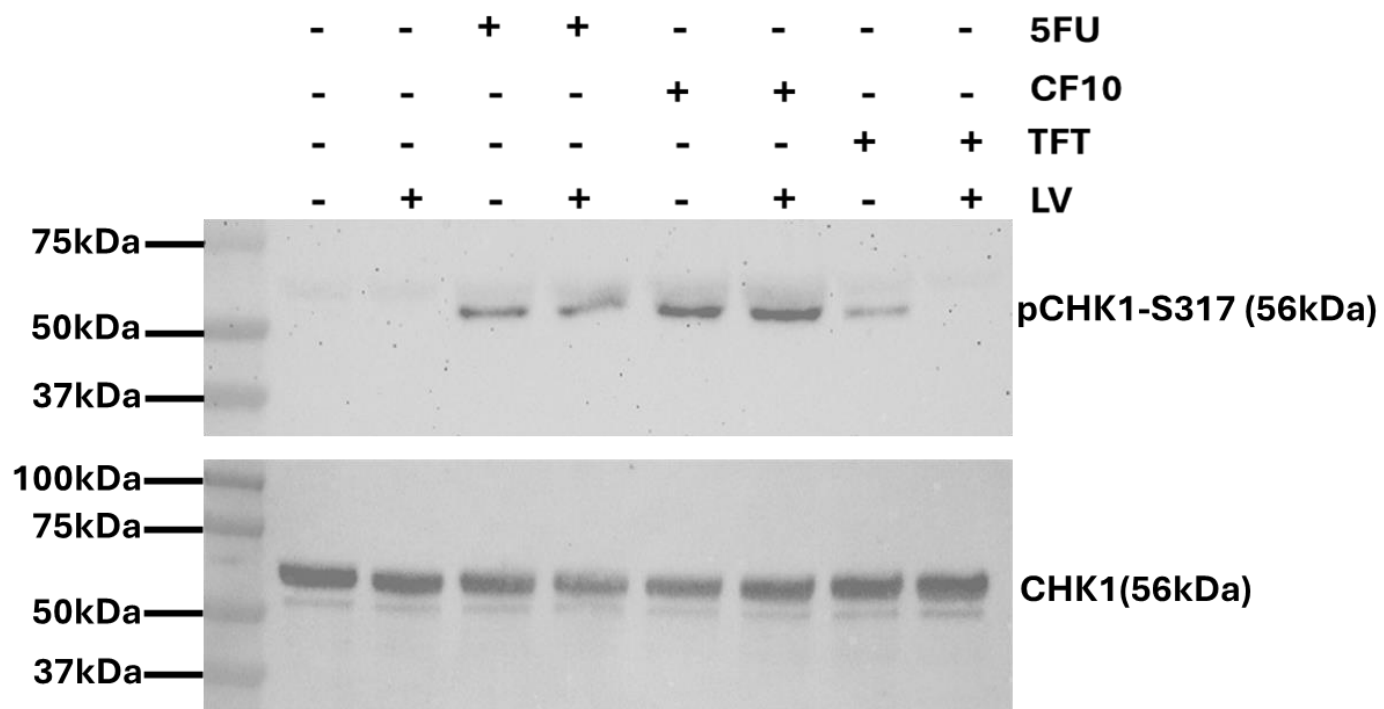

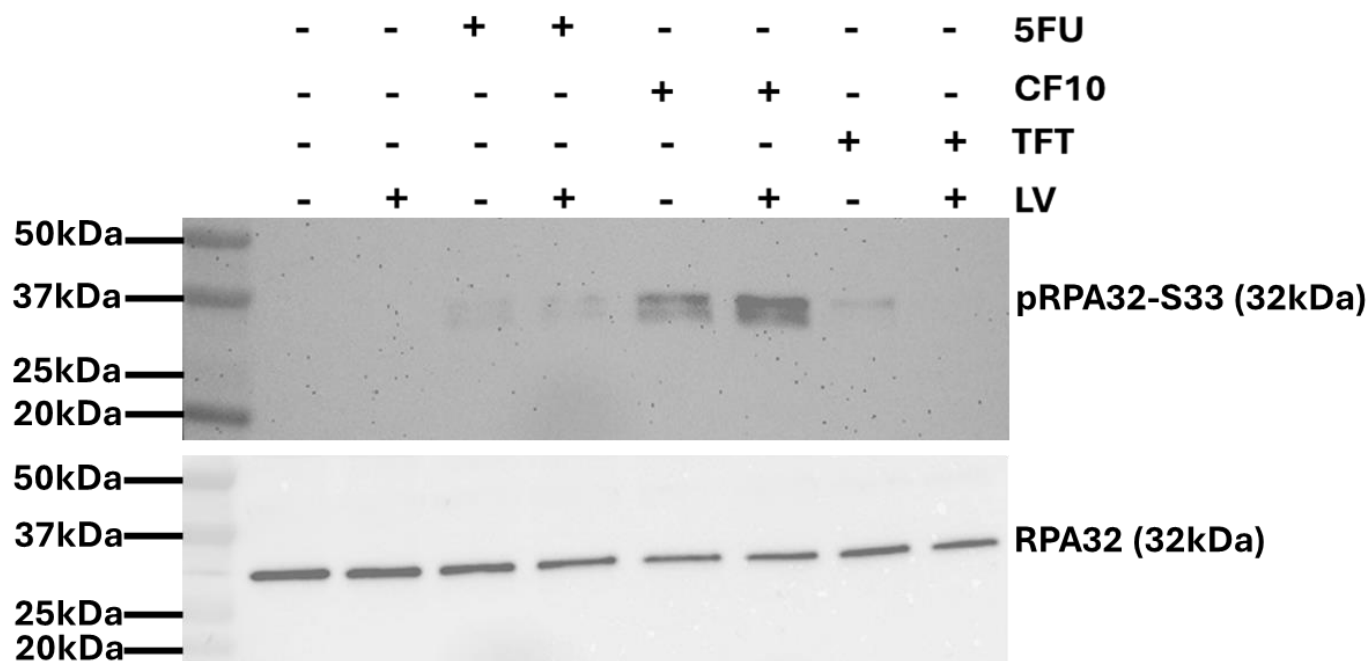

|   |   |   |   |   |   |   |   |      |
|---|---|---|---|---|---|---|---|------|
| - | - | + | + | - | - | - | - | 5FU  |
| - | - | - | - | + | + | - | - | CF10 |
| - | - | - | - | - | - | + | + | TFT  |
| - | + | - | + | - | + | - | + | LV   |

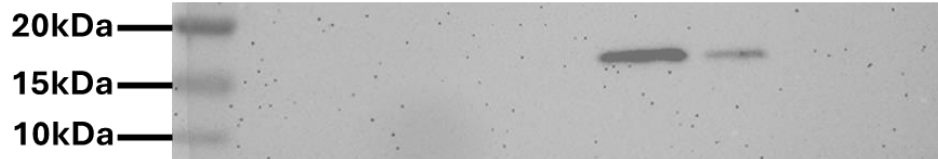

**Cleaved Caspase 3-D175  
(19kDa)**

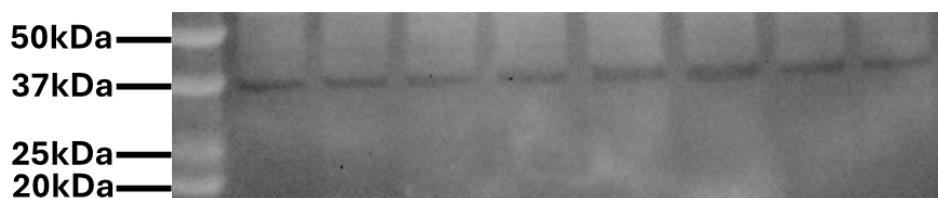

**Rad51(37kDa)**

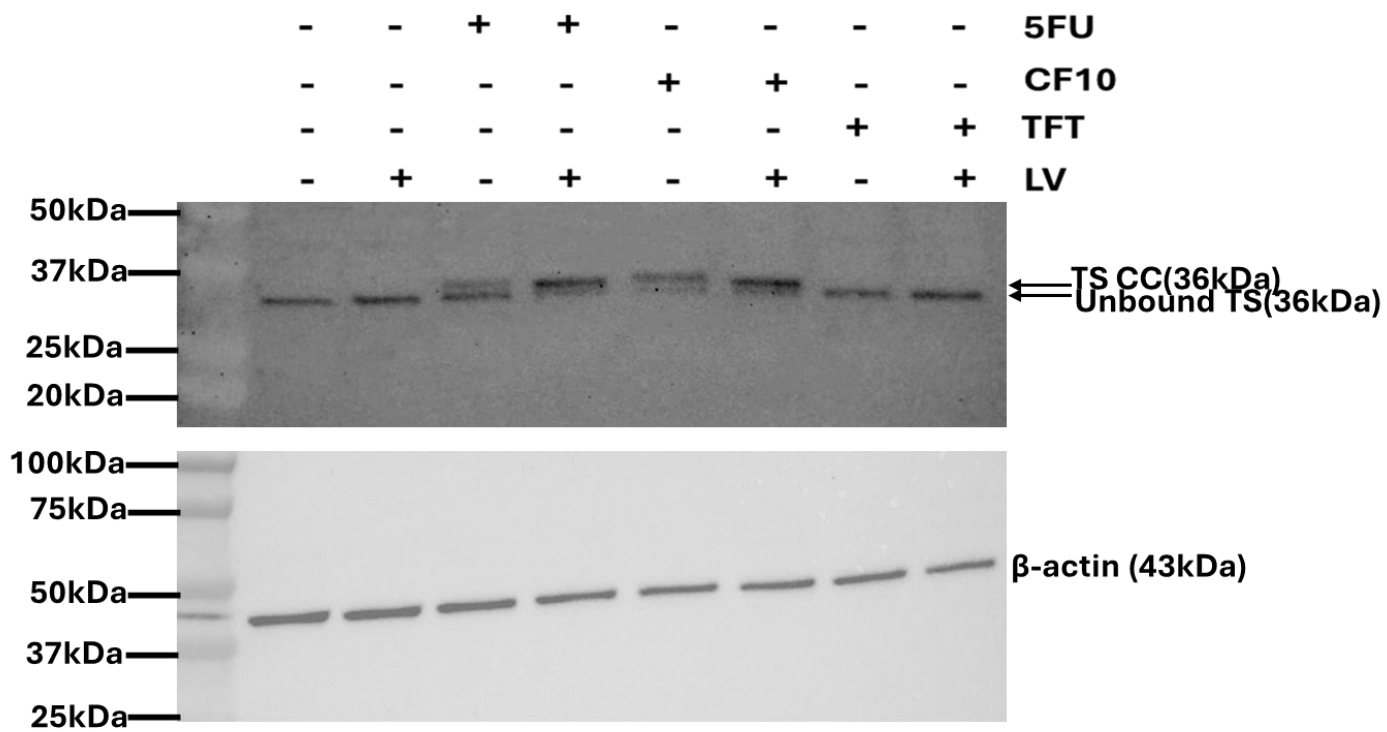

Supplement: Supplementary file 1 [file cancers-17-02739-s001.zip › cancers-3743016 Original Western Blot Images.pdf]
